# Electrophoretic Deposition of Quantum Dots and Characterisation of Composites

**DOI:** 10.3390/ma12244089

**Published:** 2019-12-07

**Authors:** Finn Purcell-Milton, Antton Curutchet, Yurii Gun’ko

**Affiliations:** 1 School of Chemistry, Trinity College Dublin, University of Dublin, Dublin 2, Ireland; antton.curutchet@ens-lyon.fr; 2 BEACON, Bioeconomy Research Centre, University College Dublin, Dublin 4, Ireland

**Keywords:** electrophoretic deposition, quantum dots, photoanode, sensitization, dichloromethane

## Abstract

Electrophoretic deposition (EPD) is an emerging technique in nanomaterial-based device fabrication. Here, we report an in-depth study of this approach as a means to deposit colloidal quantum dots (CQDs), in a range of solvents. For the first time, we report the significant improvement of EPD performance via the use of dichloromethane (DCM) for deposition of CQDs, producing a corresponding CQD-TiO_2_ composite with a near 10-fold increase in quantum dot loading relative to more commonly used solvents such as chloroform or toluene. We propose this effect is due to the higher dielectric constant of the solvent relative to more commonly used and therefore the stronger effect of EPD in this medium, though there remains the possibility that changes in zeta potential may also play an important role. In addition, this solvent choice enables the true universality of QD EPD to be demonstrated, via the sensitization of porous TiO_2_ electrodes with a range of ligand capped CdSe QDs and a range of group II-VI CQDs including CdS, CdSe/CdS, CdS/CdSe and CdTe/CdSe, and group IV-VI PbS QDs.

## 1. Introduction

Electrophoretic deposition (EPD) is a relatively new technique finding application in the field of nanotechnology, despite it having been utilised for a number of years in important areas such as the ceramic industry [1,2]. Of special significance, is the application of EPD in regards to colloidal nanoparticle based devices, with diverse examples including Au based photoanodes [3], as well as fluorescent Cu and Au nanosheet based light emitting diodes (LEDs) [4] being demonstrated as of late. In particular, the field of colloidal quantum dots (CQDs) has shown a range of noteworthy EPD applications including EPD based CQD purification [5], formation of heterojunctions [6], and light-emitting device fabrication [7].

In addition, it has been shown that photoanodes of quantum dot sensitised solar cells (QDSSC) [8] can be efficiently produced using various sizes of colloidal quantum dots (CQDs) and the EPD technique, enabling a significant improvement of photovoltaic characteristics [9]. The EPD approach utilises an electrical field to drive the deposition of CQDs in a solution upon the surfaces of the TiO_2_ electrodes of opposite polarity. The force driving CQDs deposition in an electrical field is due to the presence of a dipole and/or surface charge in CQDs, whose origin is due to several interactions. The effects of an electric field have been extensively studied in regards to CQD solutions, leading to the detection of a permanent dipole in CQDs and the origin of which is a matter of debate [10,11] Following on from this, the source of charging in CQDs has been affiliated to a number of process producing in most solutions a near isoelectric composition of positively and negatively charged CQDs.

Firstly, CQDs will generally not be stoichiometric in composition and therefore be rich in either an anion or cation species, which consequently produces CQDs rich in either cations or anions on its surface [12,13,14,15]. Hence, considering CdSe CQDs, it can be postulated that excess Cd will produce a positively charged surface, while excess Se will produce a negatively charged surface, though in both cases, the charge will be balanced via counterions or charged ligands to some level. Secondly, the presence or absence of charged ligands or counter ions in general can also have a marked effect upon the overall charge of CQD. Finally, the thermodynamic approach termed thermal charging, a process by which an equilibrium exists between the majority non-charged CQD population and a minority of charged CQDs has been used to explain the consistently equal concentrations of negative and positively charged CQDs in solutions in some nonpolar solvents [16].

The overall effects of these processes allow for the deposition of the charged CQDs upon the TiO_2_ electrodes, without the need for a ligand exchange. This avoids issues of CQD surface trap formation during the ligand exchange process [17], the presence of which cause overall reduction in luminesce of ligand exchanged CQDs and also is non-ideal for PV applications. This approach has also demonstrated the advantageous result of better penetration of the porous TiO_2_ electrode with the CQD sensitisers, producing a greater loading, and therefore higher incident photon absorption. The CQDs are also deposited close to the TiO_2_/Transparent conducting oxide junction, reducing efficiency losses affiliated to charge transport in the TiO_2_ layer. Other advantages relative to the linker approach are drastically decreased sensitization times using electrophoretic deposition (≈1–2 h vs. ≈1–2 days).

A number of approaches have been reported for CQD electrophoretic deposition, either as a method to produce a monolayer upon TiO_2_ [9,18] or as a route to deposit a thick film of CQDs [19,20], up to a micron in thickness on a range of materials. The production of monolayers has been achieved generally with the application of high voltages, to CQDs in non-polar solvents, (toluene, and hexane) [18,21,22]. The exact type of surface ligands present on the CQDs, the precise stoichiometry used in the synthesis, the size of the CQDs, the stability of the colloidal solution and the concentration of ligands in solution when attempting deposition all play a role in the results obtained when depositing monolayers [12]. Interestingly, some EPD approaches result in the deposition occurring on both the positive and negative electrode [9], while in other cases, deposition occurs much more strongly on one electrode than the other. Concentrations used when carrying out these depositions range from 10 ^−5^ to 10 ^−7^ M of CQDs, while deposition times vary hugely from 10 min to 4 h. A range of voltages are required, ranging from 50 V to 2 kV to cause CQD deposition and is strongly dependent upon the surface area of electrodes and the spacing between electrodes among other properties.

Several different types of colloidal CQDs have been deposited using EPD including CdSe CQDs [9,23], CdTe CQDs, PbS CQDs [24], PbSeS CQDs [24], CdSSe CQD [21], CdSeTe CQDs [25], CuInS_2_ CQDs [26] CuInS_2_/ZnS CQDs [27], and CdSe/CdS nanorods [18]. In addition, CQDs with various stabilizing ligands such as TOPO [9], tetradecylphosphonic acid [18] and oleic acid [20,21,28] have been deposited by EPD. While this wide range has been achieved, in most cases, the precise conditions vary widely.

The goals of this work are to demonstrate the universality of this approach to any solution of CQDs. Here, we explore the optimization of the EPD technique for production of various II-VI CQDs sensitised TiO_2_ composites including the effects of the nature of solvent, deposition time, measured current, etc. We show for the first time, the specific effectiveness of dichloromethane for the purposes of EPD of CQDs over already reported nonpolar solvents such as hexane and toluene and demonstrate the photosensitisation of nanoparticulate TiO_2_ electrodes with a range of core and core shell CQDs by EPD.

## 2. Materials and Methods

### 2.1. Materials

SnO_2_/F (Fluorine doped tin oxide, FTO) coated glass, 2.3 nm thickness, 13 Ω/sq, Na_2_S (sodium sulphide, 97%); TiCl_4_ (titanium (IV) chloride, ≥ 99%); ZnCl_2_ (Zinc chloride, 99.999%; were supplied by Sigma-Aldrich (St. Louis, MO, USA). TiO_2_ paste, (90-T Transparent paste consisting of 20 nm anatase particles and WER2-O Reflector Titania Paste) were supplied by Dyesol (Queanbeyan, Australia). All solvents used for EPD were HPLC grade and supplied by Sigma-Aldrich (St. Louis, MO, USA).

### 2.2. Synthesis of Colloidal Quantum Dots

All CQDs tested were synthesised in house using methods adopted from literature with details given in ESI section I, and detailed characterisation carried out in a previous publication [29].

### 2.3. Fabrication of TiO_2_ Working Electrode

Firstly, FTO glass was cut, cleaned and a bulk layer of TiO_2_ was deposited upon it, details of this are given in ESI section II. Following this, TiO_2_ electrodes were produced in house using a manual screen printer by firstly depositing a sourced sol of TiO_2_ (Dyesol 90-T Transparent paste consisting of 20 nm anatase particles and WER2-O Reflector Titania Paste consisting of 400 nm particles nanoparticles) which was deposited upon the bulk TiO_2_ layer. This was done using a manual screen printer purchased from A.W.T. World Trade Inc. (Chicago, IL, USA, [30] using custom screens, which were purchased from Serigraf Ltd. (Leinster, Ireland) with a 90 T polyester mesh, and were designed to print 1 × 3 cm electrodes. Two distinct types of electrodes were produced using the screen printer, Electrode A = 4 layers of 20 nm sized TiO_2_ particles, and Electrode B = 4 layers of 20 nm sized TiO_2_ particles followed by a fifth layer of light-scattering 200 nm sized TiO_2_ particles. Multiple layers were produced by heating the electrodes to 125 °C for 6 min in a furnace between depositions. After depositing the desired layers of TiO_2_ upon the electrodes, they were placed into a tube furnace and put through a pre-programmed temperature cycle (see ESI, Figure S1). This involved setting the furnace initially to 125 °C, and with inputting a heating rate of 8 °C/min. Upon commencement, the furnace heated to 350 °C and held at this temperature for 15 min, after which, it heated further to 450 °C, held for 15 min and then heated to 500 °C and held for a final 15 min. After this, the furnace switched off and the electrodes slowly cooled to room temperature. These electrodes were then ready for sensitisation. Electrodes characterization is provided in ESI section II with photo (Figure S2A,B) and diagram of structure (Figure S2C), UV-Vis absorption (Figure S3), scanning electron microscope (SEM) (Figures S4 and S5) and transmission electron microscopy (TEM) (Figure S6).

### 2.4. Electrophoretic Deposition Process

CQDs were electrophoretically deposited using a solution of dichloromethane (DCM) and the optimum CQD concentrations, which ranged between 1 × 10^−6^ M and 1 × 10^−5^ M dependent upon the exact size of the CQDs. These solutions were produced from a concentrated stock solution of CQDs in toluene, which was diluted to the desired volume and then immediately EPD deposition was carried out. Two electrodes were then immersed into the CQD solution, one a TiO_2_ electrode on FTO glass detailed above, a second of clean FTO glass (cut to the exact width and length of the TiO_2_ layer screen printed 1 × 3 cm, upon the FTO glass), separated by a 1 mm spacer. The voltage applied was 250 V and the optimum deposition time was between 15 and 30 min depending upon a sample and the space between electrodes was 1 mm. After which, quantum dots were found to have deposited upon both electrodes, producing a higher loading upon the negative electrode. The experimental set up for EPD is presented in electronic supporting information (ESI), Figure S7, with a TiO_2_ before and after EPD shown in ESI, Figure S8. Additionally, it was found that the FTO counter electrode needed effective cleaning after each deposition with the use of concentrated aqua regia, followed by washing with deionised water and isopropanol, to maintain the repeatability, during multiple deposition cycles.

### 2.5. Successive Ionic Layer Adsorption and Reaction (SILAR) Method for ZnS Coating of TiO_2_ Electrode

A ZnS layer was deposited using a modified procedure previously reported dip coating technique [31]. Firstly, an aqueous solution of 0.1 M Zn(OAc)_2_ and 0.1 M Na_2_S were produced. The working electrode was firstly submerged for 1 min into the Zn^2+^ solution (0.1 M Zn(OAc)_2_) after which, it is washed with deionised water. Following this, it was then submerged into the S^2−^ solution (0.1 M Na_2_S) for 1 min. This constituted a single cycle, depositing a layer of ZnS, to produce the desired thickness of ZnS; the cycle was carried out three times.

### 2.6. Instrumentation

Photoresponsivity measurements were carried out using a three-electrode arrangement made using a Metrohm µAutolab type III electrochemical impedance analyser (Metrohm Autolab B.V., Utrecht, The Netherlands), using a reference, counter and working electrode in a 0.1 M aqueous solution of Na_2_S using a 20 mL quartz cuvette, a white LED ring as a light source, with further details given in ESI, Section IV. Photoresponsivity Measurements, with diagram given in Figure S9 and explanation of readings in Figure S10 and emission spectra of white light LED in Figure S11. UV-Vis absorption spectroscopy was carried out using an Agilent Cary 60 UV-Vis spectrometer (Agilent, Santa Clara, CA, USA), measuring the electrodes in the air following the washing of excess solution. For all studies using UV-Vis absorption to monitor EPD deposition, the TiO_2_ electrode did not include the additional scattering layer of 400 nm TiO_2_ nanoparticles, since this strongly reduced the transparency of the electrode as shown in the ESI (see Figures S2A and S3) and therefore, were only used in the case of open cell measurements or otherwise specifically stated. HRTEM High resolution transmission electron microscopy (HRTEM) and scanning transmission electron microscopy (STEM) was carried out using a FEI Titan Transmission Electron Microscope (FEI Company, Hillsboro, OR, USA) operating at 300 kV, and SEM was carried out using a Zeiss Ultra plus SEM in the CRANN Advanced Microscope (Zeiss, Oberkochen, Germany) Facility-Trinity College Dublin. 

## 3. Results and Discussion

### 3.1. EPD of CdSe CQDs

**Figure 1:**
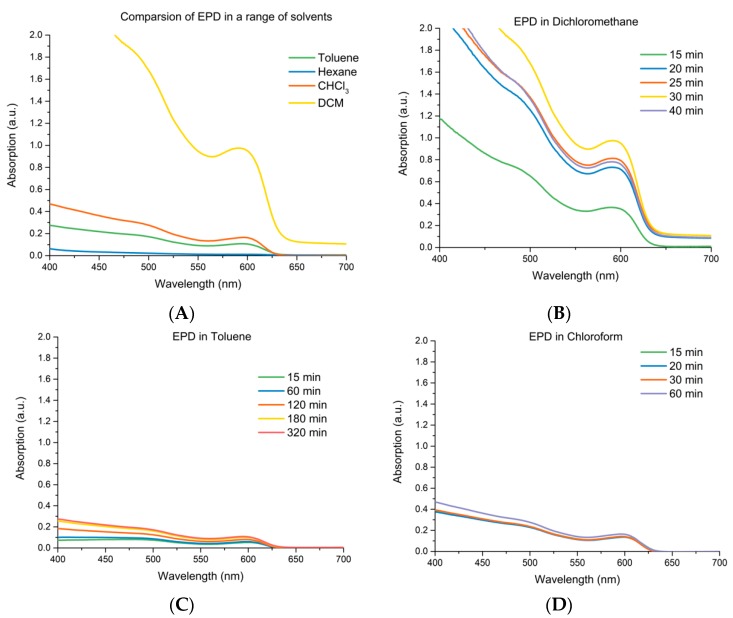
UV-Vis absorption spectroscopy was used to measure the deposition of 4.4 nm CdSe quantum dots upon a 12-µm TiO_2_ electrode. Different solvents were used with the same concentration of 2.5 × 10^−6^ M and the absorption of the films was periodically measured during the deposition. (**A**) compares the electrophoretic deposition (EPD) maximum absorption achieved for the four solvents tested, (**B**–**D**) shows the time evolution for dichloromethane (DCM), Toluene, and Chloroform, with hexane showing no time dependency.

Solvent plays a key role in the EPD rate and character of produced CQD films, with studies already demonstrated in toluene [9,21], hexane [23] and chloroform [28] to date. Therefore, for this study, we examined this effect in detail, choosing non-polar solvents in which the long chain aliphatic ligand capped CQDs were fully stable, indicated by the absence of turbidity or precipitation of samples occurring prior to deposition. The solvents with different dielectric constants were also selected to demonstrate its effect upon EPD. Therefore, four common solvents were compared to examine their effectiveness, hexane, DCM, CHCl_3_ and toluene and were investigated by examining the absorption of oleic acid capped CdSe CQDs (4.4 nm) using EPD with respect to time. After deposition, electrodes were examined using UV-Vis absorption spectrometry and the spectra are shown in Figure 1. Maximum absorption was produced on the positive electrode, with the order of absorption across the solvents given as DCM > CHCl_3_ > toluene > hexane, while time taken to reach the maximum was in the order: toluene > CHCl_3_ > DCM with hexane showing no change over time. DCM strongly outperformed all other solvents tested, showing maximum absorption after just 30 min of deposition. Therefore, DCM was chosen as the optimal solvent to carry out the further electrophoretic deposition studies.

The origin of this stark difference in EPD performance can be related to the properties of the solvents tested. Firstly the connection between solvent and the total weight deposited in EPD is given by a relationship that was first derived in 1999 [1,32] and is a modification of the Hamaker’s equation [33] (see ESI, Equation (S1)). The term which is related to the solvent properties is the electrophoretic mobility (see ESI, Equation (S2)) and relates the mobility of the particles in a solution to the medium’s permittivity, the zeta potential of the particles and an inverse relationship to the solvent viscosity. The viscosity of solvents tested is as follows, Toulene—0.59, CHCl_3_—0.536, DCM—0.413 and Hexane—0.30 and therefore does not explain the trend observed. Zeta potential is strongly dependent upon the presence of charged species such as ions or impurities when concerning non-polar solutions [34] and therefore reliable prediction of possible zeta potential trend related to solvent is not possible, though it is related to the dielectric constant and has been found to be of higher values in higher dielectric constant solvents in some studies [35]. Instead, we propose the order of EPD success can be explained by the trend in dielectric constants of these solvents, which is highest for DCM (8.93—DCM, 4.8—chloroform, 2.38—toluene, 1.88—hexane) [36]. The dielectric constant (sometimes called the ’relative permittivity’) is the ratio of the permittivity of the given dielectric to the permittivity of a vacuum, so the greater the polarization developed by a material in an applied field of given strength, the greater the dielectric constant will be. Due to the higher dielectric constant of the solvent, there exists a stronger ability of the electrode to attract CQDs out of the solution. The increase in dielectric constant also has the effect of screening charge more effectively between CQDs which has been postulated to have the effect of increasing the population of charged CQDs due to thermal charging [16]. Originally, when looking at literature values, other solvents were also considered for testing, specifically, 1,2-dichlorobenzene and 1,2-dichloroethane, which show dielectric constants of 9.93 and 10.36, respectively, and still relatively low dipole moments (C_2_H_4_Cl_2_, 1.83 D and C_6_H_4_Cl_2_ 2.14 D), meaning the nonpolar ligand capped CQDs should show some solubility [36]. However, due to the higher viscosities of these solvents relatively to DCM (DCM = 0.44 cP, C_2_H_4_Cl_2_ = 0.79 cP, and C_6_H_4_Cl_2_ = 1.32 cP,) and lower CQD solubility in them, it was judged any benefit due to the increased dielectric constants would be lost due to the reasons mentioned. In addition, apart from EPD efficiency considerations, these solvents also show a marked increase in toxicity relative to DCM [37,38].

Finally, to confirm the proposed explanation for improved DCM performance is due to increased dielectric constant, the electrophoretic mobility of CdSe CQDs in the variable solvents needs to be tested, which therefore would enable the zeta potential to be measured, and consequentially determine if the possible explanation of performance difference between the solvents is in fact due to change in zeta potential of the QDs in the solvent. This measurement has been attempted for this study, but due to the instrumentation limitations, the results obtained did not give repeatable and therefore reliable results to report, therefore this will form the basis of future investigations.

### 3.2. Effects of EPD Duration upon Total Loading

**Figure 2:**
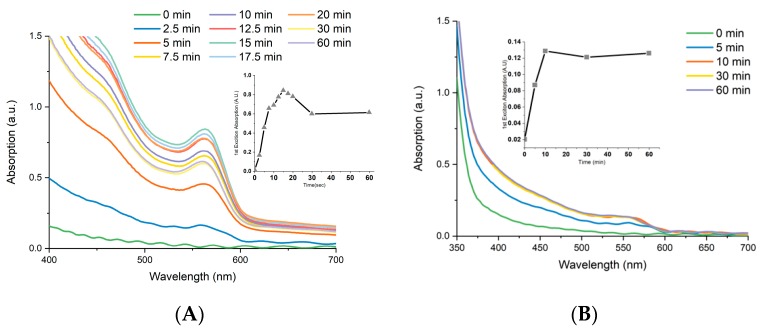
UV-Vis Absorption spectra showing the change in absorption of oleic acid capped CdSe colloidal quantum dots (CQDs) (3.3 nm) over time into a TiO_2_ electrode, showing the negative electrode (**A**) and the positive electrode (**B**), with inset depicting the change of absorption at first exciton peak.

To further analyse the behaviour of CdSe EPD in the optimal solvent, DCM, a more detailed study was carried out measuring absorption spectra at the positive and negative using TiO_2_ electrodes for each, with the results shown in Figure 2 using a 3.3 nm CdSe CQD sample. After examining both electrodes, the absorption is found to reach its peak value after 15 min on the negative electrode (Figure 2A), with the absorption remaining near maximum from between 12.5 and 20 min, after which a serious loss in sensitisation takes place. Interestingly, the same performance is not seen in the positive electrode (Figure 2B), which reached maximum absorption after 10 min, showing no change following this. This observation could be due to the population of negatively charged CQDs. Therefore, it was found that deposition time was an important factor to reaching maximum sensitisation, with too long a deposition time producing as much a negative effect as too short. The exact reason for greater sensitisation of the negative electrode is not clear, but we propose it is due to the molar cadmium excess (2.75:1, Cd:Se) used in the synthesis of the CdSe CQDs, which will produce an excess of cadmium cations in the CQDs. This was investigated by carrying out a synthesis of oleic acid capped CdSe CQDs with Se molar excess (1:2, Cd:Se), which were then electrophoretically deposited. Analysis of the resulting electrodes showed a near identical deposition upon the positive and negative electrode, which points towards the fact that stoichiometry plays only a part in the process of the overall charge present upon the CQDs, as has been reported in the literature [12].

It should also be noted that a current can be measured on the power source used to apply the voltage for EPD, which was found on average to be between 60 µA/cm^2^ and 25 µA/cm^2^ at the beginning of deposition and slowly decreased by a factor of 2 or 3 over the time of deposition. This current has been reported in literature to be caused by the movements of CQDs in solutions and the presence of impurities in either the CQD solution or solvents [12]. We observed that the current profile was the same for successful CQDs and unsuccessful CQDs depositions alike. In addition, it was noted that higher current profiles were achieved from CdSe CQD solutions, which had been put through a fewer number of cleaning cycles. From these two observations, we infer that the current is being carried by other species in solution and has no connection to EPD of the CdSe CQDs.

### 3.3. Repeatability

Interestingly, it was found that solutions diluted with DCM and immediately electrophoretically deposited produced the expected results, while solutions left to stand for 30 min, produced a much lower deposition level, and after one hour of standing, no deposition took place from these solutions. It was also observed that some solutions of CdSe CQDs completely lost their ability to be deposited when they precipitated in storage even after the CQDs were redispersed in solution. In both cases the possible explanation for this could be due to a strong reduction in electrophoretic mobility or decrease in the sticking fraction. The number of cleaning steps applied to oleic acid capped CdSe CQDs following synthesis was also found to be an important step, a large current was recorded on the power source of over 200 µA/cm^2^, while no deposition of CQDs occurred, when the original reaction solution was tested following synthesise. After one cleaning cycle, the CQDs deposited as expected, but if a second cycle of cleaning were carried out, the CQDs again would not deposit, while currents recorded would be between 20 and 40 µA/cm^2^. It was also found that after a single deposition, even though only a minor loss in overall concentration of the solution occurred (~5%, see ESI, Figure S12), no further depositions could be carried out from this solution.

### 3.4. Adherence and Comparison to Controls

**Figure 3:**
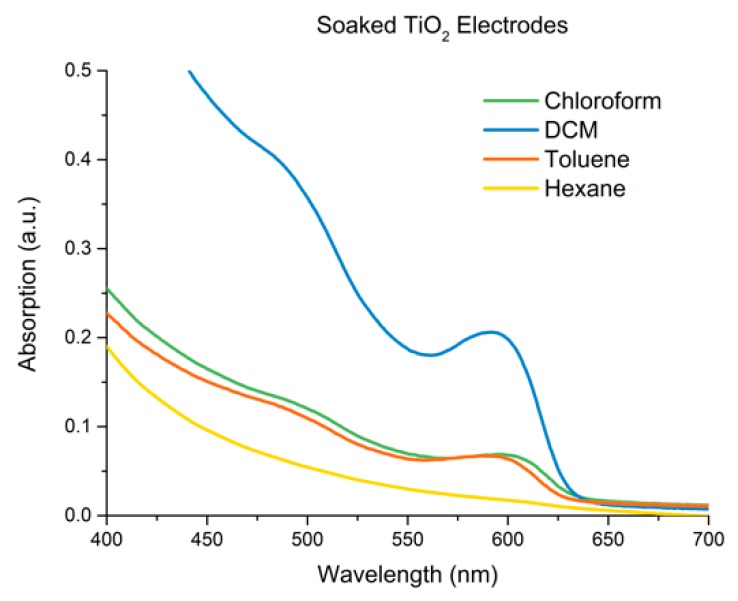
UV-Vis absorption spectrum of TiO_2_ electrode after being soaked in four different solutions of oleic acid capped CdSe CQDs at a concentration of 1 × 10^−5^ M for 3 days, using the solvents, Chloroform (black line), DCM (red line), Toluene (blue line) and Hexane (pink line).

After deposition, electrodes were washed with DCM. Interestingly, the electrodes showed only a moderate change in absorption after washing (~20%, see ESI, Figure S13) indicating the majority of CQDs were strongly bound to the surface. Following this, the efficiency of the optimised electrophoretic approach was compared against two other methods of sensitization such as soaking and drop casting. To test soaking, TiO_2_ electrodes were immersed in a CdSe solution for 3 days in darkness with a concentration of 1 × 10^−5^ M CdSe CQDs. These soaked electrodes were tested in hexane, DCM, CHCl_3_ and toluene, the same nonpolar solvents tested for electrophoretic deposition. The resulting UV-Vis spectra are shown in Figure 3. The overall absorptions were very poor, with DCM displaying the greatest sensitisation ability among these solvents. Further measurements of electrodes were carried out after 7 days of soaking, with no increase occurring. Interestingly, it should be noted that the higher performance of DCM may be related to the greater ability of DCM to penetrate the pores of the TiO_2_ electrode, and therefore may contribute to the overall improvement of DCM EPD performance. Drop casting was also tested and was carried out using a concentrated solution (1 × 10^−4^ M) of CdSe CQDs in DCM. After applying 1 mL of the solution to the electrode, it was left to dry in darkness. Following this the electrode was washed with DCM to remove excess CQDs, from this the majority of the deposited films was removed. The remaining layer that was produced was visibly uneven and patchy, showing only barely detectable CdSe absorption.

### 3.5. Electron Microscopy Analysis

**Figure 4:**
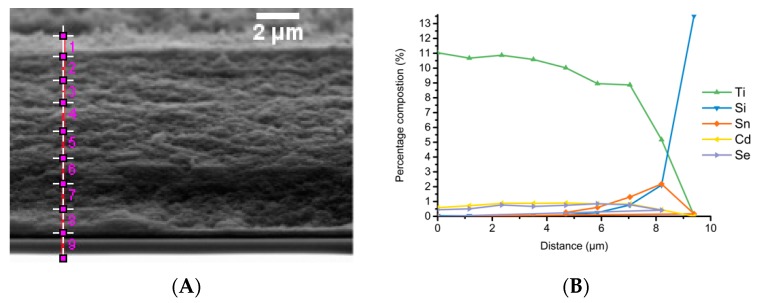
SEM and EDX of CdSe sensitised TiO_2_ electrode through electrophoretic deposition. SEM of a side on profile of the electrode is shown in image (**A**), this is marked with the positions that energy disperse X-ray spectroscopy (EDX) spectra were taken. Graph (**B**) show the elemental composition changes across the electrode as a function of distance.

Scanning electron microscopy (SEM) was used to analyse the resulting sensitised electrodes, with SEM (Figure 4A) showing no clear sign of deposition of CQDs when compared to non-sensitised electrodes (see ESI, Figures S4 and S5) indicating the absence of large CQD aggregates. In contrast, energy disperse X-ray spectroscopy (EDX) in conjunction with SEM, demonstrates the presence of CdSe QDs, via analysis of the elemental composition of the TiO_2_ films after CQD deposition, as shown in Figure 4B, enabling the CdSe CQD distribution to be determined. Using this, it was possible to establish the extent of loading of CdSe CQDs by comparing the intensity of the peak of Ti to the peaks of Cd and Se, giving a value of 7.3% Cd and 5.6% Se relative to 87.2% Ti (see ESI, Figure S14 for EDX spectra). This also allowed mapping of the elemental composition as a function of depth into the TiO_2_ film, which showed that the distribution of CdSe CQDs remained constant as a function of depth in the TiO_2_ layer. This is the optimal distribution of the sensitiser since the overall aim is to produce a near monolayer coating of CQDs upon the TiO_2_ particle surface to produce maximum increase in photon harvest followed by effective charge injection into the TiO_2_ electrode.

**Figure 5:**
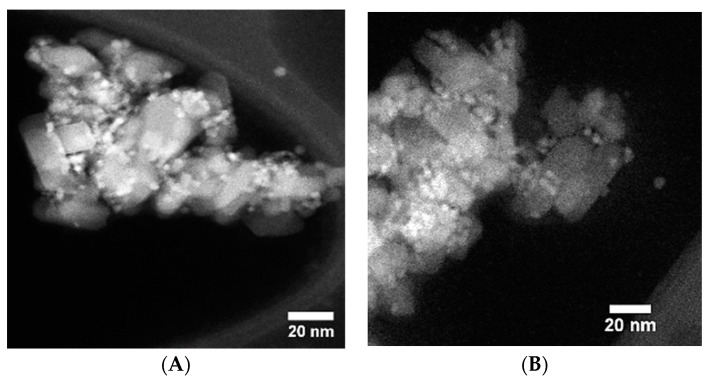

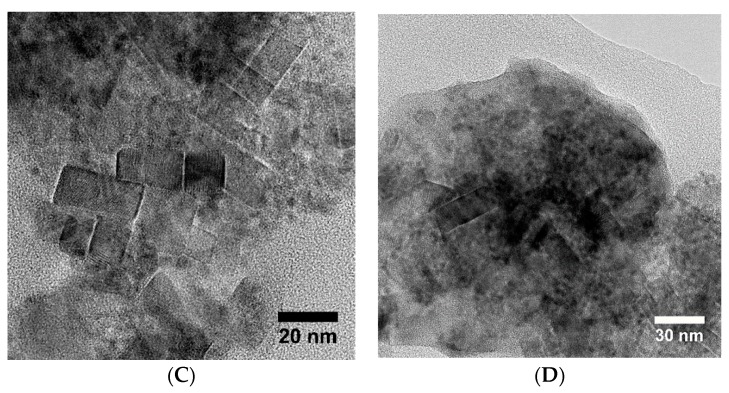
Electron Microscopy of CdSe/TiO_2_ Electrode produced using EPD, with red circles highlighting the CdSe CQD positions with STEM images (**A**,**B**) of 3.2 nm CdSe CQDs upon the surface of a 20 nm TiO_2_ NPs, while (**C**) and (**D**) show the TiO_2_ sensitised with 2.9 nm CdSe CQDs upon the surface of 20 nm TiO_2_ NPs.

Transmission electron microscopy (TEM) was then used to analyse the sensitised TiO_2_, by removing a portion of the TiO_2_ film and grinding it up in a mortar and pestle. This was then dispersed in a solution of ethanol and then drop cast onto a lacey carbon TEM grid. The resulting images are shown in Figure 5. From the images, TiO_2_ NPs are seen surrounded by a number of CdSe CQDs of 3.2 nm in diameter. Scanning transmission electron microscopy (STEM), allowed even clearer images to be produced, due to its much stronger Z-contrast as shown in Figure 5A,B. From these, the presence of individual CdSe CQDs can be seen upon the TiO_2_ NP surface and is marked in the image.

### 3.6. Photoresponsivity

Following sensitisation of the TiO_2_ photoanodes, the photoactivity of these electrodes was tested, by monitoring the photoresponsivity of the electrode using an electrochemical setup, recording the photocurrent response produced under illumination and is an approach used to analyse a range of different quantum dot sensitised electrodes for photoactivity [39,40,41,42,43].

To do this, the test was designed to allow effective examination of the resulting photocurrent response of the electrodes under illumination with two electrolytes tested, MeOH and a 0.1 M aqueous solution of Na_2_S. MeOH has been shown to act as a sacrificial hole scavenger electrolyte in QDSSCs [44], while Na_2_S is used as a vital part of polysulphide based electrolytes [45]. The current generated from testing with the MeOH electrolyte produced a very poor response of only 0.003 mA/m^2^ with our CdSe test electrode (See ESI, Figure S15A, blue line). While, using the 0.1 M aqueous solution of Na_2_S a much stronger signal was produced, giving a response of 0.035 mA/m^2^ under illumination (see ESI, Figure S15A, green line) Subsequent to this, a non-sensitised electrode of pure TiO_2_ was used to get a baseline for the response. The electrode displayed a minimal signal, producing currents up to 0.0031 mA/cm^2^ under illumination (see ESI, Figure S15B).

**Figure 6:**
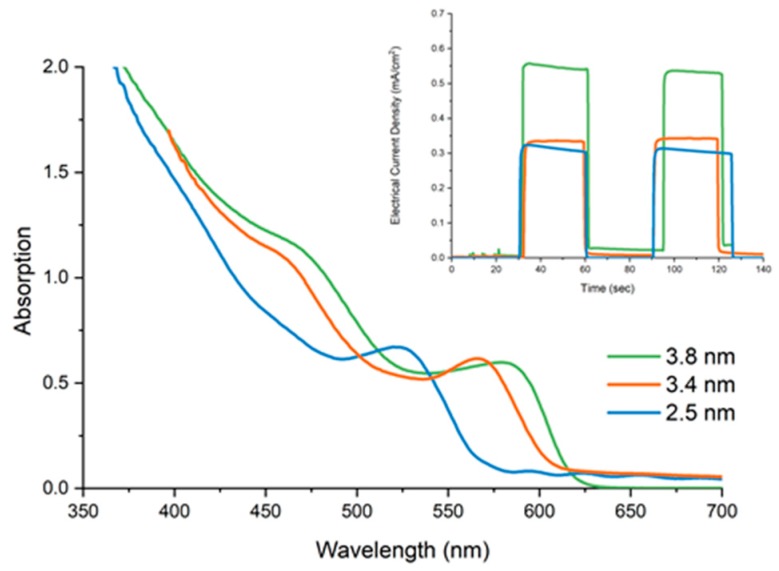
UV-Vis absorption of TiO_2_ electrodes sensitised with oleic acid capped CdSe CQDs with 2.5 nm, 3.4 nm and 3.8 nm diameters, with the inset showing the resulting photocurrent responses under illumination with illumination with 2.5 nm giving 0.31 mA/cm^2^, 3.4 nm giving 0.35 mA/cm^2^, and 3.8 nm giving 0.55 mA/cm^2^.

Following this, three different sizes of oleic acid capped CdSe CQDs, with diameters of 2.5 nm, 3.4 nm and 3.8 nm were deposited upon TiO_2_ electrodes and the photocurrent was tested, as shown in Figure 6. This was performed to demonstrate that variables size of nanoparticles can be easily deposited, with it additionally displaying the effect of CdSe CQD size upon the produced photocurrent. It was found that as the size of the CQDs grew, an increase in current took place from 0.31 mA/cm^2^ to 0.35 mA/cm^2^ to 0.54 mA/cm^2^. This can be explained by the wider absorption of larger CQDs, and the alignment of the conduction bands therefore enabling them to harvest more photons from the incident light and effectively inject them into the TiO_2_.

### 3.7. ZnS Treatment of Electrodes

ZnS is a large band gap semiconductor, and has been shown to form a type I band gap alignment with Cd chalcogenides [46,47] and has been reported as a means to increase the photocurrent of QDSSCs [48]. The reason for this increase is twofold, firstly the ZnS coating produces a type I band alignment which reduces recombination loss in CQDs, secondly due to the coating approach, it also coats the TiO_2_ surface, acting to reduce recombination loss in the TiO_2_ electrode [49]. The ZnS deposition was performed using the SILAR approach and used three cycles of Zn and S deposition to produce the resulting layer using 0.1 M aqueous solutions of sodium sulphide and zinc acetate.

**Figure 7:**
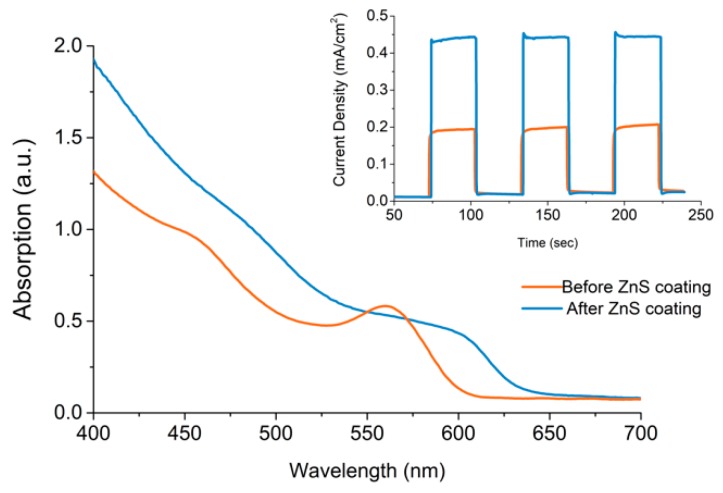
UV-Vis absorption spectroscopy, comparing the change in absorption of an oleic acid capped CdSe CQD (3.4 nm) sensitised TIO_2_ electrode due to ZnS coating with inset showing the resulting increase in photocurrent upon illumination.

UV-Vis absorption spectroscopy was used to show the change in absorption of a CdSe sensitised TiO_2_ electrode and is shown in Figure 7. A significant increase in absorption also occurs when observing the CdSe CQD sensitised TiO_2_ electrode, which also produces a large red shift in the absorption of the CdSe CQD also, which is due to the lattice mismatch between the materials that effects the electronic structure of the CdSe CQD. Finally, photocurrent action response was used to then analyse the resulting change in photocurrent response of a CdSe sensitised TiO_2_ electrode under illumination and is shown in Figure 7. The resulting electrodes shows an increase in photocurrent due to ZnS coating, increasing from 0.19 mA/cm^2^ in the absence of ZnS to 0.44 mA/cm^2^ after ZnS coating.

### 3.8. Open Cell Measurements

**Figure 8:**
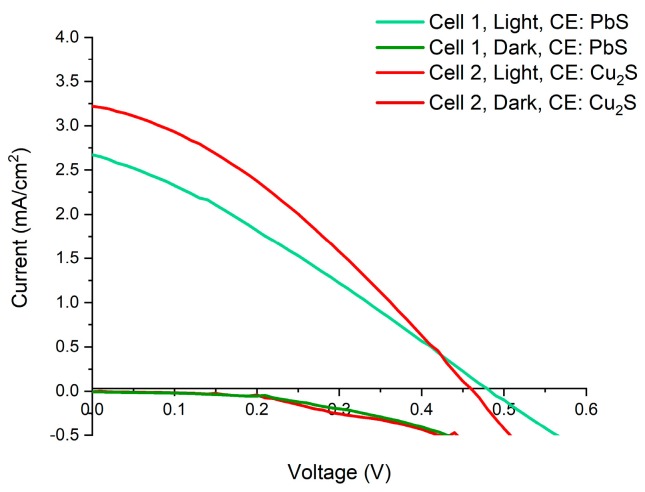
IV-curve of open cell measurement of QDSSC, using oleic acid capped, 4.3 nm, CdSe CQDs. The open cell measurements were made using two different counter electrodes, composed of either a Cu_2_S electrode on brass foil or a PbS electrode on Pb foil.

To further characterise the photoanodes, open cell measurements were carried out which give an indication of possible performance if incorporated into a QDSSC design (see ESI, VI. Open Cell Measurements). Briefly, IV measurements were carried out which involved immersing the photoanode and counter electrodes into a polysulphide electrolyte solution (an aqueous solution of 2 M S and 2 M Na_2_S) under illuminated with an AM 1.5 Xe Arc Lamp (see ESI, Figure S16). The counter electrodes used were fabricated in house with details and characterisation given in ESI (PbS, Figures S17 and S18) and Cu_2_S (see ESI, Figures S19 and S20). Tests were carried out using OA-capped 4.3 nm CdSe CQD sensitised TiO_2_ and ODPA capped 5.1 nm CdSe CQD sensitised TiO_2_ using either a PbS or Cu_2_S counter electrodes, with the best results shown in ESI. Table S1 and the IV curve graph of the OA capped CdSe sensitised photoanode shown in Figure 8, producing a PCE of 0.501%, giving a V_oc_ = 0.46, an I_sc_ = 3.22, and a FF = 33.8% for Cu_2_S counter electrode and producing a PCE of 0.38%, giving a V_oc_ = 0.48, an I_sc_ = 2.67, and a FF = 29.82% for PbS counter electrode. These results clearly show the effectiveness of this EPD approach in producing efficient and active photoanodes.

### 3.9. Demonstration of EPD with a Range of Alternatively Ligand Capped CdSe CQDs and Other Core and Core/Shell CQDs

In addition, EPD of CdSe CQDs capped in an alternative ligand shell were also tested, since these CQDs generally show higher luminescence in solution, and therefore better surface passivation, an aspect previously analysed by ourselves on these CQDs [29], and could play an important characteristic in reducing recombination when sensitising the TiO_2_ electrode. In addition, we want to confirm the universality of the developed EPD approach. The CdSe CQDs investigated were octadecylamine capped (ODA) and octadecylphosphonic acid (ODPA) capped CdSe with resulting absorption and photocurrent response given in the ESI (Figure S21 for ODA capped and Figure S22 for ODPA capped). In both cases, the deposition was successful and shows the potential of this approach for a range of ligand capped CQDs.

Following this we confirmed the ability to deposit CQDs of larger or smaller bandgaps than CdSe, enabling the exact widow of sensitivity to be tuned by CQD choice, therefore CdS CQDs, a UV absorber and PbS CQDs, a NIR absorber were also deposited using EPD. The results of these is given in the ESI (see Section VII). CdS shows effective sensitization in the UV range, confirmed using UV-Vis absorption and photoresponsivity (Figure S23), and uniform distribution across the electrodes mapped using SEM and EDX (Figure S24). PbS shows the same effective sensitization but across the visible spectrum to NIR, confirmed using UV-Vis absorption and photoresponsivity (Figure S25), and also the same uniform distribution across the electrodes mapped using SEM and EDX (Figure S26).

Finally, following on from this work, core/shell CQDs were also tested to understand if this approach could be used with a wider range of more complex CQDs, which have been reported to produce even more efficient QDSSC designs [31] and for use in a range of other important applications. Therefore core/shell CQDs of CdSe/CdS, CdS/CdSe and CdTe/CdSe were separately EPD upon TiO_2_ electrodes, the results of which are given in the ESI (see Section VII). For each sample strong deposition too place (Figure S27) and were confirmed using photoresponsivity and UV-Vis absorption (CdSe/CdS, Figure S28; CdS/CdSe, Figure S29 and CdTe/CdSe, Figure S30). This results again demonstrates the wide applicability of the approach developed here. Therefore, though our study used CdSe CQDs as a model system to understand the effectiveness of EPD, this work underlines the wide range of important CQDs that can be easily deposited using this approach.

## 4. Conclusions

In conclusion, in this work, we have demonstrated the wide applicability of EPD as an approach to sensitise TiO_2_ electrodes with a diverse range of CQDs. In addition, we have demonstrated that for the first time the importance of solvent choice and how this can greatly optimise the efficiency of EPD. We propose that by choosing a solvent with enhanced dielectric constant and one which is still an effective solvent of choice for long alkyl chain ligand capped CQDs, EPD loading can be greatly improved. In addition, it is important to stress that due to limitations of instrumentation, electrophoretic mobility and therefore zeta potential could not be confirmed for CdSe CQDs in these solvents, therefore there remains the possibility that some of the EPD performance improvement reported here could be due to zeta potential changes in these alternative solvents also, and hence this will form the basis for future investigations. In summation, the wide application of EPD for colloidal nanoparticle systems are still an approach that is underutilised, this work adds to the growing body of studies that greatly enhances its efficiency and demonstrates its huge potential.

## Supplementary Materials

The following are available online at https://www.mdpi.com/1996-1944/12/24/4089/s1, Figure S1: Electrophoretic deposition setup to deposit quantum dots upon electrodes, Figure S2: TiO_2_ Electrodes before (**A**) and after (**B**) r EPD deposition of CdSe QDs, Figure S3: This is the experimental design used to take photoresponsivity measurement the photoaniode illumination is carried out using a white light LED ring, Figure S4: This shows the ideal current response of a photoaniode when cycled between illumination/darkness as a function of time, Figure S5: Emission spectra of white light LED used to approximate sunlight illumination for photocurrent spectra measurements, Figure S6: Photos of transparent TiO_2_ electrode (**A**) produced from 4 layers of screen printed 90T Dyesol 20 nm TiO_2_ paste, and the same electrode (**B**) incorporating a 5th light scattering layer consisting of sintered 200nm TiO_2_ particles and (**C**) a diagram of the Produced electrodes show the different layers present, Figure S7: SEM images of nanoparticulate TiO_2_ surface on FTO glass. Image A is looking down on the film, and shows a continuous TiO_2_ coating, with no visible cracks. This SEM also shows the overlap of three TiO_2_ coatings. Image B shows a cross section of a 4-layer TiO_2_ electrode which shows a continues film, measuring 12 µm in thickness, Figure S8: This UV-Vis absorption spectra of a FTO coated glass with a differing number of porouse TiO_2_ layer deposted upon its surface. The spectra shows the change in absorption as the number of TiO_2_ layers are depopsted while also expressiones the excellent transpancy of this film to visible absorption even with 4 layers which equates to a thikness of between 10–12 µm of TiO_2_, Figure S9: SEM image (**A**) of side profile of a TiO_2_ electrode. consisting of 10 µm thick layer, of 20 nm sintered TiO_2_ nanoparticulate layers, (**B**) is a top view SEM image of a 3 µm thick scatter layer consisting of 200 nm sintered TiO_2_ nanocrystals, Figure S10: Image (**A**) shows the UV-Vis absorption spectra of TIO_2_ electrode with scatter layer, demonstrating the large absorption of these electrodes. Image (**B**) shows the photocurrent response of CdSe QD (4.4 nm) sensitised TiO_2_ showing the effect of the inclusion of the scatter layer producing an increase from 0.56 mA/cm^2^ to 0.71 mA/cm^2^ producing a 27% increase in current, Figure S11: TEM images of the 20 nm TiO_2_ nanoparticulate after sintering. Image (**A**) shows a number of the TiO_2_ crystals, while image (**B**) shows the lattice fringes produced from these samples under HRTEM examination, showing a spacing of 0.165 nm which matches to the (211) lattice plane of anatase TiO_2_, Figure S12: UV-Vis absorption spectroscopy of oleic acid capped CdSe (3.5 nm) sensitised TiO_2_ electrode. Comparing the change in loading of QDs when the electrode is washed with DCM following EPD deposition of CdSe QDs, showing a loss of 20 % of loading due to the wash, Figure S13: UV-Vis absorption spectroscopy of oleic acid capped CdSe (3.8 nm) in DCM showing the change in solution concentration due to deposition. Which shows a loss of ~5.5% in concentration when comparing the concentration of before and after deposition, Figure S14: EDX of CdSe sensitised TiO_2_ electrode through electrophoretic deposition, EDX spectra produced from position 5 on the SEM image, showing the elemental loading of QDs relative to Ti, Figure S15: Photocurrent action response (**A**) of a oleic acid capped CdSe QD sensitized TiO_2_ electrode using MeOH (red line) or 0.1 mol aqeouse solution of Na_2_S (black line) as an electrolyte. Photocurrent response (**B**) of TIO_2_ electrode without QD sensitization, which produced only a minimal current of 0.0031 mA/cm^2^ under illumination, Figure S16: Photocurrent action response of ODA capped CdSe QDs showing a peak current of 0.25mA/cm^2^ from 4.2 nm CdSe QDs, Figure S17: UV-Vis absorption spectroscopy (**A**) and the resulting photocurrent action response (**B**) of ODPA capped CdSe QDs (5.2 nm) EPD deposited upon a TiO_2_ electrode, Figure S18: Photos of the original Pb Foil and the PbS counter electrode produced from this, Figure S19: SEM images of the three stages in material as the originally purchased Pb foil (image **A**) is firstly converted to PbSO_4_ (image **B**) and then to PbS (image C). To produce the desired PbS counter electrode. Graph D and E shows the elemental composition of the PbS film determined using EDX spectroscopy, Figure S20: Photos of original brass foil and the produced Cu_2_S coated foil produced from this, Figure S21: SEM images of brass foil before and after treatment to produce the Cu_2_S electrode. The elemental composition of the foils was examined using EDX and compared to the original films described in the spectra and pie charts of both, Figure S22: The emission spectra of the Xenon arc discharge lamp used for solar simulation, Figure S23: This shows the UV-vis absorption and the resulting photocurrent action response of a TiO_2_-FTO electrode sensitized with 3 nm CdS QDs. **A**) UV-Vis absorption shows the original QD spectra in solution and then the resulting spectra of the electrodes sensitized. The positive and the negative electrode both show depostion, with higher laoding taking place upon the postive elctrode. **B**)Photocurrent action spectra shows a on-off response under illumination. 0.01127mA/cm^2^, 0.0053mA/cm^2^, Figure S24: SEM and EDX of a CdS QD sensitised nanoporous 4 µm TiO_2_ electrode with 3 nm CdS QDs. (**A**) Shows SEM of the side profile of TiO_2_ electrode on FTO glass, with an EDX line profile overlaid on the image showing the relative abundance of different elements in this electrode. (**B**) EDX line profile of elemental composition while (**C**) is the EDX line profile just showing the distribution of just QD related materials, Cadmium and Sulphur across the electrode. Image **D** shows relative loading of QDs related materials in the TiO_2_ electrode at depth of 2 µm relative to titanium while image E shows the EDX spectrum of the electrode at a depth of 2 µm in the TiO_2_ electrode, Figure S25: UV-Vis absorption spectra (**A**) and resulting photocurrent response (**B**) of PbS QD sensitised TiO_2_ electrode. (**A**) Shows the resulting absorption of an electrophoretically deposited PbS (2.7 nm) QD upon a TiO_2_ electrode. B shows the resulting photocurrent response under illumination, producing a peak current of 0.13 mA/cm^2^, Figure S26: This shows the sensitisation of a nanoporous ~ 17-µm TiO_2_ electrode with 2.7 nm PbS QDs. (**A**) Shows a SEM of the side profile of one of these electrodes with an EDX line profile overlaid on the image showing the relative abundance of different elements in this electrode. (**B**) EDX line profile of elemental composition. (**C**) EDX line profile show distribution of just QD materials across the electrode. (**D**) Elemental comparison of QDs in the TiO_2_ electrode at depth of 7.5 µm. (**E**)EDX spectrum of the electrode at a depth of 7.5 µm in the TiO_2_ electrode, Figure S27: Photo of TiO_2_ electrodes sensitised by CdS/CdSe QDs, CdTe/CdSe QDs, and CdSe/CdS QDs, Figure S28: UV-Vis absorption spectra and photocurrent response (inset) of CdSe/CdS sensitised TiO_2_ electrode. The total photocurrent produced was 0.05 mA/cm^2^, Figure S29: UV-Vis absorption spectra CdTe/CdSe QD sensitised TiO_2_ electrode and photocurrent response (inset) of the resulting electrode measured under illumination producing a current of 0.316 mA/cm^2^, Figure S30: UV-Vis absorption spectra and photocurrent response (inset) of CdS/CdSe sensitized TiO_2_ electrode, giving a photocurrent of 0.57mA/cm^2^, Table S1: This shows the result of open QDSSC cell testing under AM 1.5, 1 sun illumination, showing the resulting characteristics of the cells determined by measuring the IV response of the produced cells.
