# Supplementary material for: Electrophoretic Deposition of Quantum Dots and Characterisation of Composites
Source: Materials (Basel). 2019 Dec 7;12(24):4089. doi: 10.3390/ma12244089 (PMC6947596; doi:10.3390/ma12244089)
Supplement: Supplementary file 1 [file materials-12-04089-s001.pdf]

Electrophoretic Deposition of Quantum Dots and Characterisation of Composites

Finn Purcell-Milton [1,2,*], Antton Curutchet [1] and Yurii Gun'ko [1,2,*]

[1] School of Chemistry, Trinity College Dublin, University of Dublin, Dublin 2, Ireland; antton.curutchet@ens-lyon.fr
[2] BEACON, Bioeconomy Research Centre, University College Dublin, Dublin 4, Ireland
* Correspondence: fpurcell@tcd.ie (F.P.M.); igounko@tcd.ie (Y.G.)

1. Quantum Dot Synthesis Methods

1.1. Materials

All materials were used as supplied unless stated otherwise.

1.1.1. Materials Supplied by Sigma-Aldrich

Acetone (HPLC grade), SnO_2 /F (Fluorine doped tin oxide) coated glass, 2.3 nm thickness, 13 Ω/sq, OA (oleic acid, 90%); ODA (octadecylamine, 97%); ODE (octadecene , ≥97%); OLY (oleylamine, 70%); Rhodamine 6G (99%); Se (99.99%); %); TBP (tributylphosphine, 90%); Terpineol (95%, mixture of isomers); TDPA (tetradecylphosphonic acid, 97%); TOP (trioctylphosphine, 90%); TOPO (trioctylphosphine oxide, 99%).

1.1.2. Solvents Supplied by Trinity College Solvent Stores

Acetone, chloroform, dichloromethane ethanol, propanol, hexane and toluene

1.1.3. Other Suppliers

Cd(stearate)$_2$, (min 90%, Strem Chemicals); HPA (n-hexylphosphonic acid, 97%, ABCR); ODPA (n-octadecylphosphonic acid, ≥97%, PCL Synthesis); S (99.999 %, Acros Organics).

1.2. Preparation of Oleic Acid Capped CdS Quantum Dots

This work was carried out using a modified method from literature. [1,2]

1.3. Preparation of 0.05 M Sulphur Stock Solution

0.016 g (0.5 mmol) of sulphur was dissolved in 10.0 mL of degassed ODE, produced by heating the solution under argon to 200 °C for 15 min.

1.4. CdS QD Synthesis

0.0128 g (0.997 mmol) of CdO, 0.093 g of OA (0.33 mmol) and 3.9 g of ODE were weighed into a 100 ml 3-neck round-bottom flask equipped with a condenser and thermometer. The solution was heated to 30 °C and degassed for 20 min under vacuum. The reaction was then switched to an argon atmosphere and then heated to 300 °C to allow formation of Cd(oleate)$_2$, which was indicated by a colour change from brown to colourless. Following this 1.0 mL of a 0.05 M solution of sulphur dissolved in ODE was injected. Growth was then allowed to proceed for 10 min after which the reaction was removed from the heat source and the CdS QDs were precipitated using degassed ethanol. The QD solution was cleaned using a number of dissolution and precipitation cycles with dry hexane and HPLC grade acetone.

1.5. Preparation of Oleic Acid Capped CdSe

This work was carried out using a modified method from literature.[3]

1.6. Se-TBP Solution Preparation

0.1105 g (1.399 mmol) of Se was mixed with and 3.0 mL of TBP under an argon atmosphere and sonicated until the Se become completely dissolved, indicated by a clear transparent solution.

1.7. CdSe QD Synthesis

0.50 g (0.0039 mol) of CdO, 4.0 g of oleic acid and 10.0 g of ODE (octadecene) were weighed into a 100 ml 3-neck round-bottom flask equipped with a condenser and thermometer. Following this, the flask was put under vacuum and degassed for 20 min at 30 °C. After this, the flask was switched to argon; the temperature was increased to 260 °C and allowed to stabilize. Time was then allowed for the CdO to convert to $Cd(oleate)_2$ which was indicated by a colour change from brown to colourless. At this point, the Se-TBP solution was injected. Growth was allowed to proceed for a set amount of time (10–220 s). The reaction was then removed from the heating mantle and allowed to cool to 200 °C, following by injection of 20 mL of degassed acetone to precipitate the QDs. The resulting QD solution was cleaned using a number of dissolution and precipitation cycles with dry hexane and HPLC grade acetone.

1.8. Preparation of ODPA Capped CdSe QDs

This work was carried out using a modified method from literature [4].

1.9. Preparation of Se 0.75 M Stock Solution

0.059 g of Se was mixed with 1.0 mL of TOP under an argon atmosphere and sonicated until the Se become completely dissolved, indicated by a clear transparent solution.

1.10. Synthesis of CdSe QDs

TOPO (3.0 g), ODPA (0.280 g) and CdO (0.060 g) were added to a 100 mL 3-neck round-bottom flask equipped with a condenser and thermometer. The solution was then heated to 150 °C and degassed under vacuum for 30 min. The atmospheres was then changed to argon and the solution was heated to 300 °C and held at this temperature until the solution turned optically clear and colourless, indicating formation of $Cd(ODPA)_2$. Following this 1.0 mL of TOP was injected into the flask; the solution was then heated to the required temperature. The Se-TOP solution was then injected and immediately the solution was removed from the hot plate and the reaction mixture was allowed to cool. After the solution cooled to below 150 °C, 20 mL of dried methanol was injected to precipitate the NCs and was left to cool to room temperature. The resulting QD solution was cleaned using a number of dissolution and precipitation cycles with dry toluene and methanol.

1.11. Preparation of Octadecylamine Capped—CdSe QDs

This work was carried out using a modified method from literature [5].

1.12. Preparation of 1.5 M Se Stock Solution

1.184 g of Se was mixed with 10.0 mL of TOP under an argon atmosphere and sonicated until the Se become completely dissolved, indicated by a clear transparent solution.

1.13. Preparation of 0.5 M Cd Stock Solution

A 0.5 M solution of Cd was created by firstly degassing a solution of 1.285 g of CdO in 10.0 mL of OA and 10.0 mL ODE, which was then switched to an argon atmosphere and heated to 270.0 °C, until a clear solution was created.

1.14. SILAR Stock Solutions of Cd and Se

To grow the CdSe QDs further, we used a solution of 0.2 M Se stock solution and 0.2 M Cd stock solution, diluted using solvents mentioned above.

1.15. Synthesis of CdSe QDs

7.50 g of octadecylamine, 2.50 g of TOPO, 20 g of octadecene, and 2.0 mL of 0.5 M Cd-oleate were added to a 100 mL 3-neck round-bottom flask equipped with a condenser and thermometer. This solution was heated to 80.0 °C and degassed under vacuum for 20 min. Following this, the solution was switched to an argon atmosphere and heated to 290 °C. After which 1.0 mL of 1.5 M Se-TOP solution was injected and QD growth began. After injection of Se-TOP at 290 °C, the temperature was set at 250 °C for CdSe QD growth for 10 min. Following this stock solutions of Se and Cd were slowly injected in cycles to increase the size of QDs, beginning with 0.20 mL of Se, following this 0.40 mL of Cd was injected, then 0.40 mL of Se and finally 0.80 mL of Cd.

After ten minutes, the solution was cooled down to room temperature, and CdSe QDs were precipitation with acetone. The QDs were separated using centrifugation and cleaned using a number of cycles of hexane dissolution and acetone precipitation followed by centrifugation. CdSe core QDs were then re-dispersed in hexane.

1.16. Preparation of PbS Quantum Dots

This work was carried out using a modified method from literature [6].

1.17. Preparation of 0.1 M TMS Stock Solution

0.042 ml of TMS (0.2 mmol) was mixed with 2.0 mL of degassed ODE, under argon.

1.18. Oleic Acid Capped PbS QD Synthesis

0.090 g of PbO (0.4 mmol), 0.224 g of OA and 3 g of ODE were added to a 100 mL 3-neck round-bottom flask equipped with a condenser and thermometer. The flask was put under a vacuum and degassed for 30 min while heating the solution to 30 °C. Following this, the atmosphere was changed to argon and the solution was heated to 120 °C and held there until the solution becomes colourless and transparent, indicating the complete conversion of PbO to $Pb(oleate)_2$. The solution then was brought to the desired injection temperature and the TMS stock solution was added. The reaction was allowed to proceed for 10 min after which the reaction was removed from the heat and quenched with 20 mL of HPLC grade acetone. The solution was then cleaned using a number of dissolution and precipitation cycles with dry hexane and HPLC grade acetone.

1.19. Preparation of CdS/CdSe Quantum Dot

This work was carried out using a modified method from literature[1,7].

1.20. Preparation of 0.5 M Sulphur Stock Solution

0.016 g of sulphur was dissolved in 10.0 mL of degassed ODE, produced by heating the solution under argon to 200 °C for 15 min.

1.21. Preparation of 0.4 M Se Stock Solution

A 0.4 M stock solution was prepared by dissolving 0.063 g of Se in 2 mL of trioctylphosphine using sonication under argon, producing a clear solution.

1.22. Preparation of 0.4 M Cd $(oleate)_2$ Stock Solution

A 0.4 M solution of Cd was created by firstly degassing 0.5135 g of CdO in 10.0 mL of a 1:1 (v/v) OA:ODE solution which was then switched to an argon atmosphere and heated to 270 °C, until a clear solution was created.

1.23. CdS/CdSe QD Synthesis

0.0128 g (0.00010 mol) of CdO 0.093 g of OA (0.33 mmol) and 3.9 g of ODE were added to a 100 mL 3-neck round-bottom flask equipped with a condenser and thermometer. The flask was then put

under vacuum and heated to 30 °C, degassing the solution for 20 min. The reaction was then switched to an argon atmosphere and then heated to 300 °C to allow formation of Cd(oleate)$_2$ indicated by the solution becoming clear. Following this 1.0 mL of a 0.05 M solution of sulphur dissolved in ODE was injected (0.016 g of sulphur in 10.0 mL of degassed ODE, produced by heating the solution under argon to 200 °C for 15 min). Growth was then allowed to proceed for 10 min. After which the vessel was cooled to 215 °C and shell pre-cursor injection began.

Firstly, 0.8 mL of degassed OA was added into the reaction vessel. This was followed by injection of 0.15 mL of a Se stock solution and followed by the addition of an equimolar amount of Cd(oleate)$_2$ precursor stock solution after a 10 min interval. Following this, another 10 min was allowed for the reaction to take place. This constitutes one cycle of shell deposition. Three more cycles of Se and Cd stock solutions were injected with the same 10 min intervals between each injection, increasing the volume from 0.25 mL, to 0.4 mL to 0.6 mL for each cycle. The reaction was terminated by removing the heating source room and precipitated the QDs with degassed ethanol. The solution was then cleaned using a number of dissolution and precipitation cycles with dry hexane and HPLC grade acetone.

1.24. Preparation of CdSe/CdS Core-Shell Quantum Dots

This work was carried out using a modified method from literature [5].

1.25. Preparation of 0.1 M S Stock Solution

Sulphur stock solution was prepared by dissolving 0.032 g of sulphur in 10.0 mL of degassed ODE at 180 °C under an argon atmosphere to obtain a 0.1 M sulphur solution.

1.26. Preparation of 0.1 M Cd Stock Solution

A 0.1 M solution of Cd was created by firstly degassing 0.128 g of CdO in 10.0 mL of a 1:1 (v/v) OA:ODE solution, which was then switched to an argon atmosphere and heated to 270 °C, until a clear solution was created.

1.27. Synthesis of CdSe/CdS QDs

10 g of oleylamine, 20 g of ODE, and 6.75×10^{-7} mol of octadecylamine capped–CdSe QDs (see Section CdS synthesis) were added to a 100 ml 3-neck round-bottom flask equipped with a condenser and thermometer. The flask was then put under vacuum and heated to 60 °C, degassing the solution for 30 min. Following this, the reaction was switched to an argon atmosphere and 0.3 mL of the 0.1 M Cd stock solution was injected. This was heated to 230 °C and allowed to react for 10 min. This was followed by injection of 1.25 mL of a S stock solution and followed by the addition of an equimolar amount of Cd(oleate)$_2$ precursor stock solution after a 10 min interval. Following this another ten minutes was allowed for the reaction to take place. This constitutes one cycle of shell deposition. Two more cycles of shell deposition were carried out with the same 10 min intervals between each injection, increasing the volume to firstly 2.25 mL of S stock solution and 0.45 mL of Cd stock solution and then 3.25 mL of S stock solution and 0.6 mL of Cd stock solution. The reaction was terminated by removing the heating source room and precipitated the QDs with degassed ethanol. The solution was then cleaned using a number of dissolution and precipitation cycles with dry hexane and HPLC grade acetone.

1.28. Preparation of CdTe/CdSe Type II Core-Shell Quantum Dots

This work was carried out using a modified method from literature [8].

1.29. 0.08 M Te Stock Solution

0.0127 g (0.995 mmol) of Te, 0.20 mL of TOP (Trioctylphosphine), 0.050 g (0.15 mmol) of ODPA and 1 mL of ODE were added to a sample bottle under a strict argon atmosphere. This was heated to 120 °C and remained at this temperature until the solute became fully dissolved, producing a clear

strongly yellow coloured solution, if it became opaque, oxygen contamination has taken place and the solution must be discarded and preparation must be repeated.

1.30. 0.09 M Se Stock Solution

A 0.09 M solution of 0.071 g (0.0009 mol) of Se was added to 1.6 mL of TOP, in 8.4 mL of degassed ODE under argon and sonicated to produce a clear solution.

1.31. 0.1 M Cd Stock Solution

A 0.1 M solution of Cd was created by firstly degassing 0.128 g (0.997 mmol) of CdO in 10.0 mL of solution containing 1.25 mL of OA and 8.75 mL of ODE, which was then switched to an argon atmosphere and heated to 270 °C, until a clear solution was achieved.

1.32. Synthesis of CdTe/CdSe Quantum Dots

0.026 g (0.20 mol) of CdO, 0.226 g (0.800 mmol) of OA (oleic acid) and 3 g of ODE was added to a 3-neck flask with a condenser and thermometer. This was degassed 30 °C using vacuum and then heated to 280 °C under argon, and time was then allowed for the Cd to coordinate with the OA, indicated by the solution becoming colourless. This was then followed by injection of the Te stock solution. This is allowed to react for 3 min. Following this, the reaction is cooled to 230 °C and shell precursors are added alternatively. This began by injection of 1.0 mL of Se stock solution over one minute, which was then allowed to react for ten minutes, and then followed by 1.0 mL of Cd stock solution, added over a one-minute period, which was then allowed to react over ten minutes. This process is continued, making alternative injections of shell precursors until all of the stock solutions had been added. The reaction is then cooled, and acetone is added to precipitate the QDs.

2. TiO_2 Electrode Fabrication and Characterisation

2.1. FTO Electrode Cleaning

Fluorine doped tin oxide glass was cut using a glass scribe and then cleaned. The cleaning process consisted of three stages. Firstly, the glass was submerged in deionized water and detergent for 90 min and sonicated [9], and then washed using deionized water. This was followed by sonication in ethanol for 90 min, washed with acetone and finally sonication in acetone for 90 min. After which the glass was dried in an oven at 80 °C.

2.2. Bulk TiO_2 Layer Deposition upon FTO Electrode

TiO_2 electrodes were produced by firstly depositing a bulk layer of TiO_2 upon the FTO glass using a modified reported procedure [10]. This was done by producing firstly a 2 M (4.5 mL) solution of $TiCl_4$ in 20 mL of deionised water in a 3-neck round bottom at 0 °C. This was then stored in the freezer until use. As the FTO will be used as the cathode terminal, TiO_2 will be only placed on FTO. A solution of 0.006 mL of TiO_2 in 25 mL of deionised water (0.0003 M, $TiCl_4$ solution) is injected on the FTO side of the electrode. It was heated for 30 min at 125 °C in an oven. The electrodes were washed using deionised water and ethanol followed by drying.

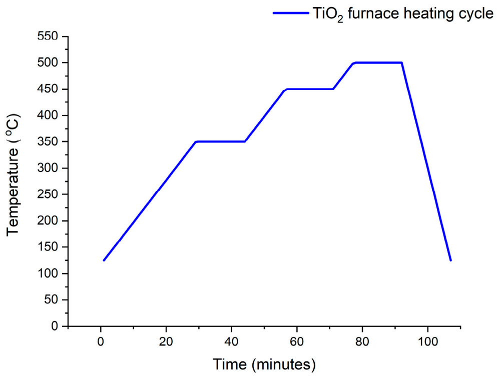

Figure S1. TiO_2 electrode thermal treatment cycle using a tube furnace in air.

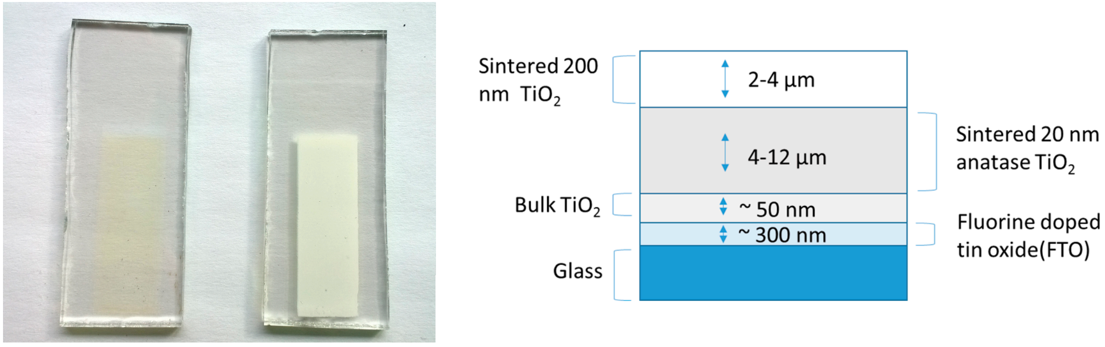

(**A**) (**B**) (**C**)

Figure S2. Photos of transparent TiO_2 electrode (**A**) produced from 4 layers of screen printed 90T Dyesol 20 nm TiO_2 paste, and the same electrode (**B**) incorporating a 5th light scattering layer consisting of sintered 200 nm TiO_2 particles and (**C**) a diagram of the Produced electrodes show the different layers present.

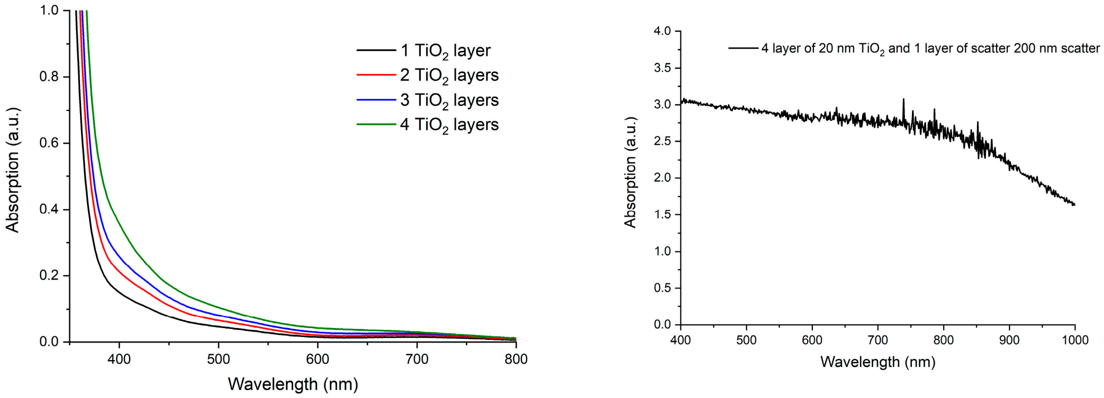

(**A**) (**B**)

Figure 3. This UV-Vis absorption spectra of a FTO coated glass with a differing number of porouse TiO_2 layer deposted upon its surface (**A**). The spectra shows the change in absorption as the number of TiO_2 layers are depopsted while also expressiones the excellent transpancy of this film to visible absorption even with 4 layers which equates to a thikness of between 10-12 µm of TiO_2 (**B**).

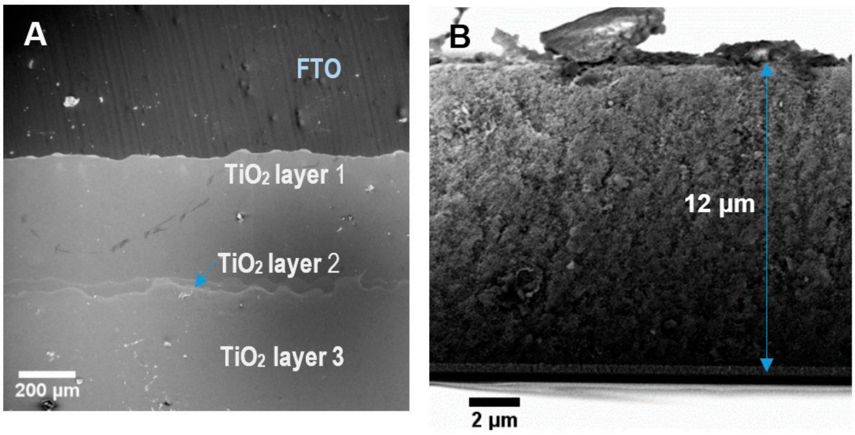

Figure S4. SEM images of nanoparticulate TiO_2 surface on FTO glass. Image A is looking down on the film, and shows a continuous TiO_2 coating, with no visible cracks. This SEM also shows the overlap of three TiO_2 coatings. Image B shows a cross section of a 4-layer TiO_2 electrode which shows a continues film, measuring 12 µm in thickness.

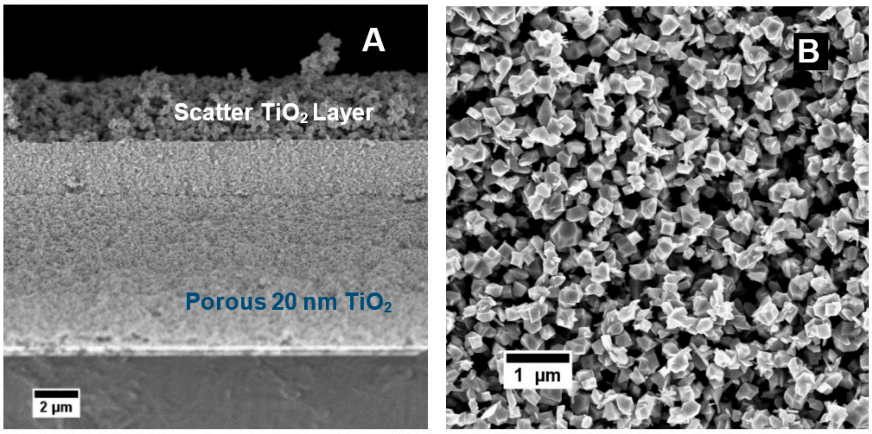

Figure S5. SEM image (**A**) of side profile of a TiO_2 electrode. consisting of 10 µm thick layer, of 20 nm sintered TiO_2 nanoparticulate layers, (**B**) is a top view SEM image of a 3 µm thick scatter layer consisting of 200 nm sintered TiO_2 nanocrystals.

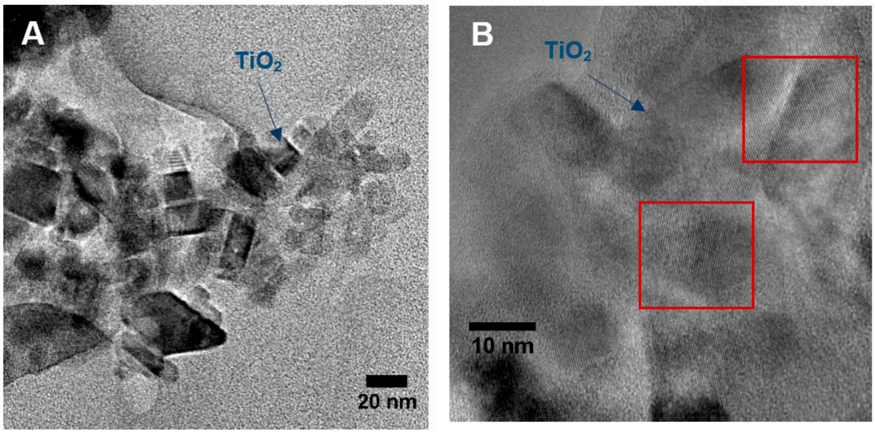

Figure S6. TEM images of the 20 nm TiO_2 nanoparticulate after sintering. Image (**A**) shows a number of the TiO_2 crystals, while image (**B**) shows the lattice fringes produced from these samples under HRTEM examination, showing a spacing of 0.165 nm which matches to the (211) lattice plane of anatase TiO_2.

3. Electrophoretic Deposition: Additional Information

This describes the factors which effect the deposition weight achieved upon a planar electrode and so accurately describes the factors to consider the deposition of QDs upon an electrode. This relationship was first derived in 1999 [11] [12] and is a modification of the Hamaker's equation [13].

$$w = -\mu E S C_d \frac{\phi_s}{\phi_d - \phi_s} t \quad \text{(S1)}$$

Where deposit yield (w), the electric field strength (E), the surface area of the electrode (S), φ_s and φ_d are the volumetric concentration of particles in suspension and deposit, respectively, C_d is the mass concentration of particles in the deposit, t is time and μ is the electrophoretic mobility and is given by

$$\mu = \varepsilon\xi/6\pi\eta \quad \text{(S2)}$$

This describes the factors that affect the electrophoretic mobility of particles in solution, in which ε is the permittivity, ξ is the zeta potential and η is the viscosity of the solution.

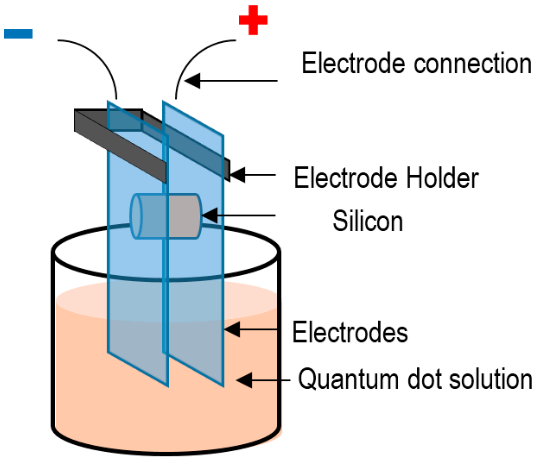

Figure S7. Electrophoretic deposition setup to deposit quantum dots upon electrodes.

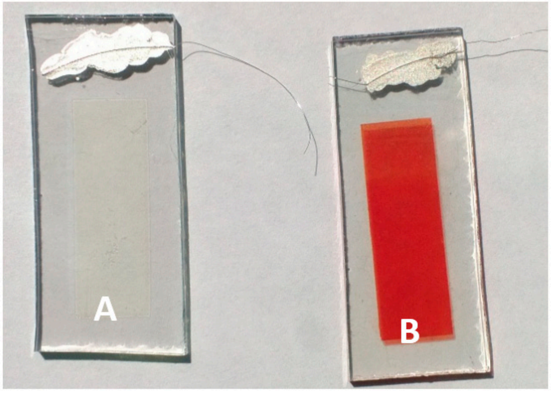

Figure S8. TiO_2 Electrodes before (**A**) and after (**B**) EPD deposition of CdSe QDs.

4. Photoresponsivity Measurements

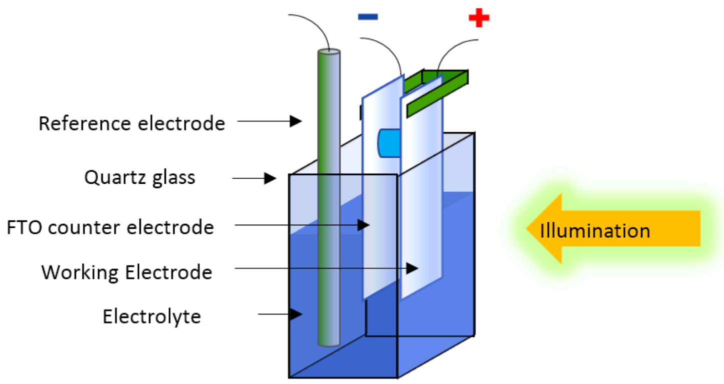

Figure S9. This is the experimental design used to take photoresponsivity measurement the photoaniode illumination is carried out using a white light LED ring.

The test was done using a three-electrode arrangement made using a Metrohm μAutolab type III electrochemical impedance analyser (Metrohm Autolab B.V., Utrecht, The Netherlands), using a reference, counter and working electrode in a 0.1 M aqueous solution of Na_2S using a 20 mL quartz cuvette (as shown in Figure S9). This was controlled using NOVA software. The reference electrode used was a saturated calomel electrode, while the counter electrode was an FTO slide. The counter electrode and working electrodes were separated using a 6 mm silicone spacer. The sample was placed 5.5 cm from the illumination source, with the working electrode illuminated through the FTO glass, which was placed parallel to the quartz glass cuvette. The working electrode was masked to produce a 1 cm^2 active area. Current readings were taken using a zero bias between the working and counter electrode and the response of the sensitised electrode was measured under darkness and illumination utilising a white light LED ring that was powered using an external voltage source, which produced on/off response of current readings when the material was photoactive (see Figure S10). The illumination approximated the visible region (see Figure S11) of the solar spectrum and allowed a determination of electrodes for the utilisation of the visible region of the solar spectrum for photovoltaic applications, the intensity of the source measured using a calibrated power meter.

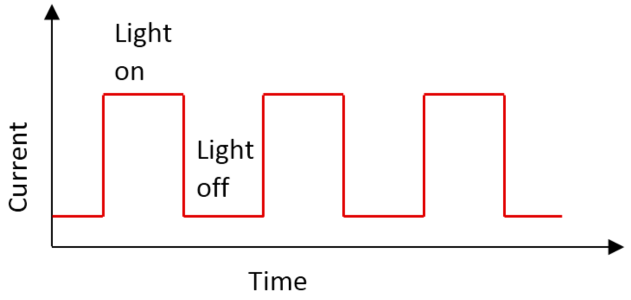

Figure S10. This shows the ideal current response of a photoaniode when cycled between illumination/darkness as a function of time.

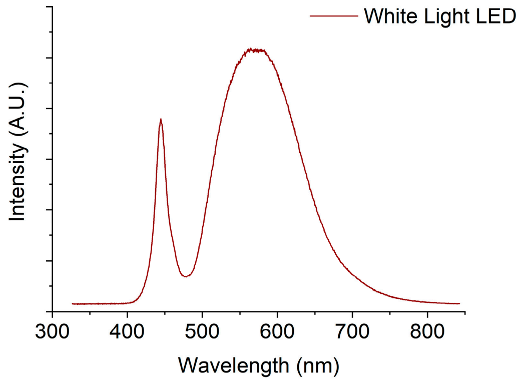

Figure S11. Emission spectra of white light LED used to approximate sunlight illumination for photocurrent spectra measurements.

5. Additional OA capped CdSe QD Electrophoretic Deposition and Post-treatment Data

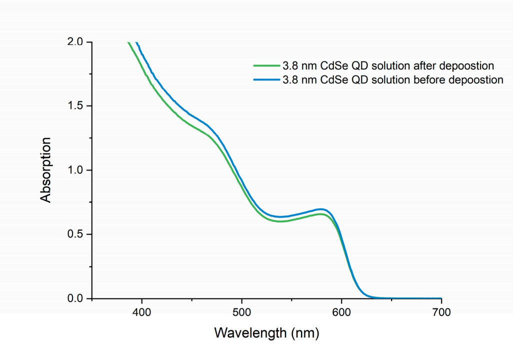

Figure S12. UV-Vis absorption spectroscopy of oleic acid capped CdSe (3.8 nm) in DCM showing the change in solution concentration due to deposition. Which shows a loss of ~5.5% in concentration when comparing the concentration of before and after deposition.

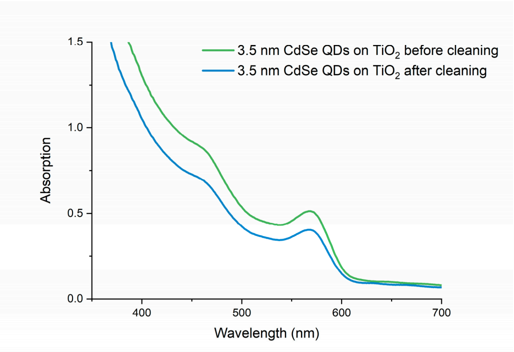

Figure S13. UV-Vis absorption spectroscopy of oleic acid capped CdSe (3.5 nm) sensitised TiO_2 electrode. Comparing the change in loading of QDs when the electrode is washed with DCM following EPD deposition of CdSe QDs, showing a loss of 20% of loading due to the wash.

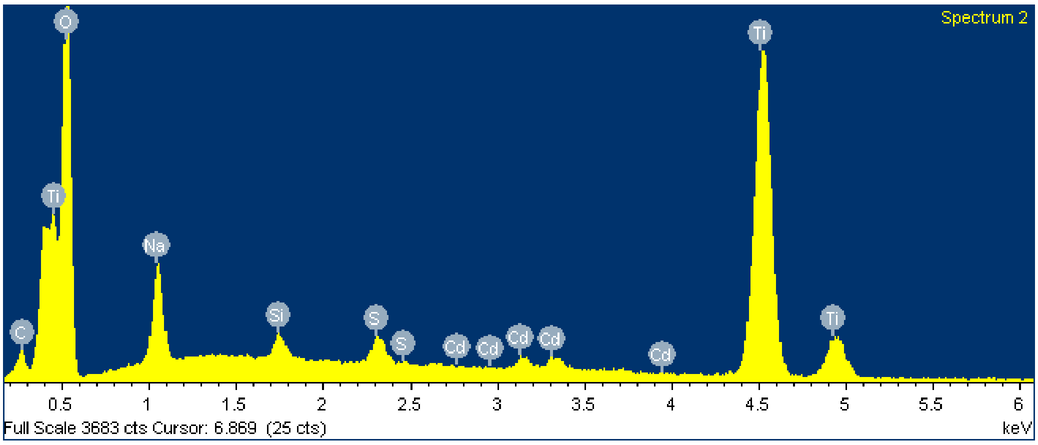

Figure S14. EDX of CdSe sensitised TiO_2 electrode through electrophoretic deposition, EDX spectra produced from position 5 on the SEM image, showing the elemental loading of QDs relative to Ti.

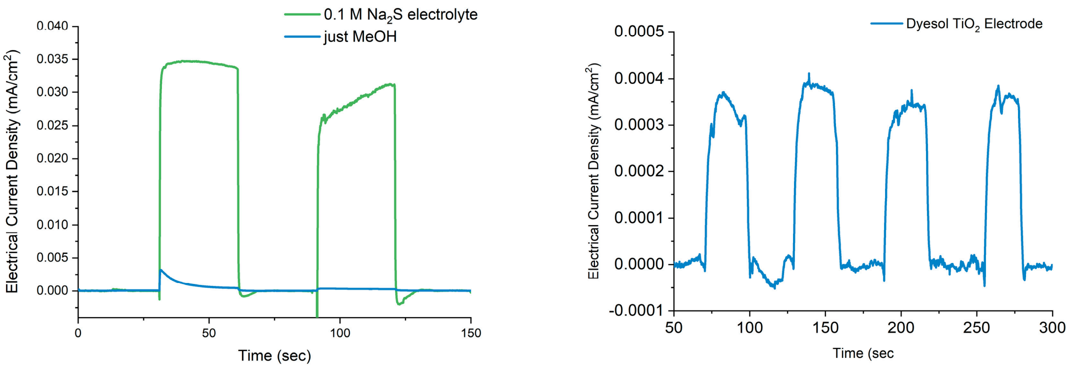

(**A**) (**B**)

Figure S15. Photocurrent action response (**A**) of a oleic acid capped CdSe QD sensitized TiO_2 electrode using MeOH (red line) or 0.1 mol aqeouse solution of Na_2S (black line) as an electrolyte. Photocurrent response (**B**) of TIO_2 electrode without QD sensitization, which produced only a minimal current of 0.0031 mA/cm^2 under illumination. 6. Open Cell Measurements

6.1. Current-Voltage (IV) Curve

The standard approach to characterise a PV cell is by IV curve analysis, comparing the cells response under illumination and under darkness. Firstly, crocodile clips were attached to the positive and negative junctions of the solar cell. These junctions were then connected to a Keithley 2400 sourcemeter (Cleveland, Ohio, United States). The light source used was a 150 W Xe-short arc lamp with a "Ozone free" coating from Ushio (uxl-150SO, USHIO AMERICA, inc. 5440 Cerritos Ave., Cypress, CA 90630), powered in an Oriel Instruments 50-500 W arc lamp housing injunction with a AM 1.5 D filter (emission spectra of lamp given Figure S16). The resulting power of this light was determined using a calibrated photodiode, to produce an illumination of 1000 W/m^2, also called 1 sun. Then through the use of a sourcemeter, Keithley 2400 sourcemeter and which was controlled through a computer using a simple program built using LabView software (LabVIEW 2013), the current readings from the cells are recorded as a voltage is swept from a negative value to a positive value. This reading is taken under illumination of the light source and under darkness and produces the resulting graph IV curve.

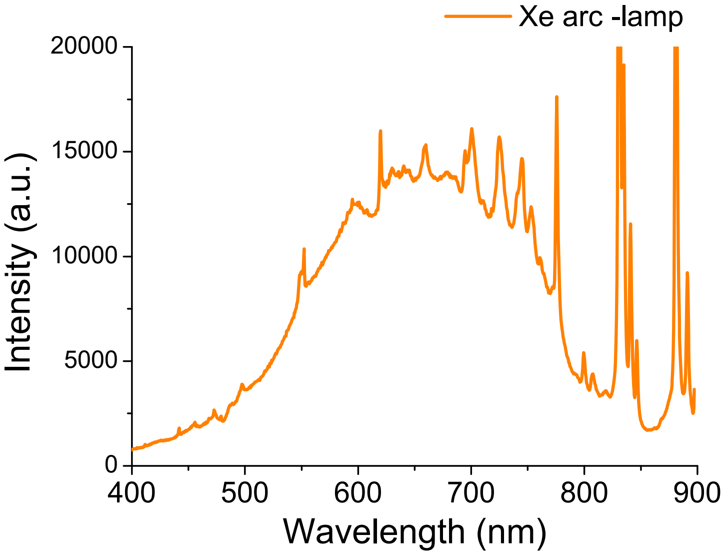

Figure S16. The emission spectra of the Xenon arc discharge lamp used for solar simulation.

6.2. Counter Electrodes

Two different counter electrodes (CE) have been fabricated and tested for open cell measurements. The electrodes examined were Cu_2S and PbS. And were characterised by EDX, SEM, with the synthetic details and characterisation given below.

6.3. PbS counter electrode

The PbS electrode is fabricated from a 0.1 mm thick Pb foil with the original foil and the produced electrode shown in Figure S17. The surface of the Pb foil was firstly converted from Pb foil to $PbSO_4$ through a treatment in 9 M H_2SO_4 at 60 °C for 1 h. The electrode was then washed with deionised water and then immersed into an aqueous solution of 1 M Na_2S and 1 M S for 24 h, producing PbS. This was then examined using SEM and EDX as shown in Figure S18. SEM was used to examine the change in surface morphology from the two step treatment carried out, showing the clear change in morphology going from the relative smooth surface Pb foil (Figure S18 A) to the roughened surface of the $PbSO_4$ (Figure S18 B) and then finally the strong change in morphology that occurred when converting to PbS (Figure S18 C). The elemental composition of the resulting PbS electrode was then examined with the use of EDX spectroscopy as shown in Figure S18E, with the elemental ratio between Pb and S shown in Figure S18D. The EDX showed the presence of sulphur in the spectra, while the resulting ratio determined was 20% S to 80% Pb ratio, indicating the formation of a PbS surface coating upon the electrode [14].

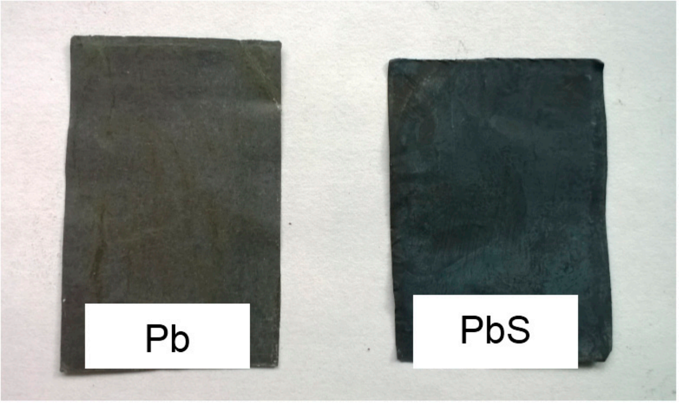

Figure S17. Photos of the original Pb Foil and the PbS counter electrode produced from this.

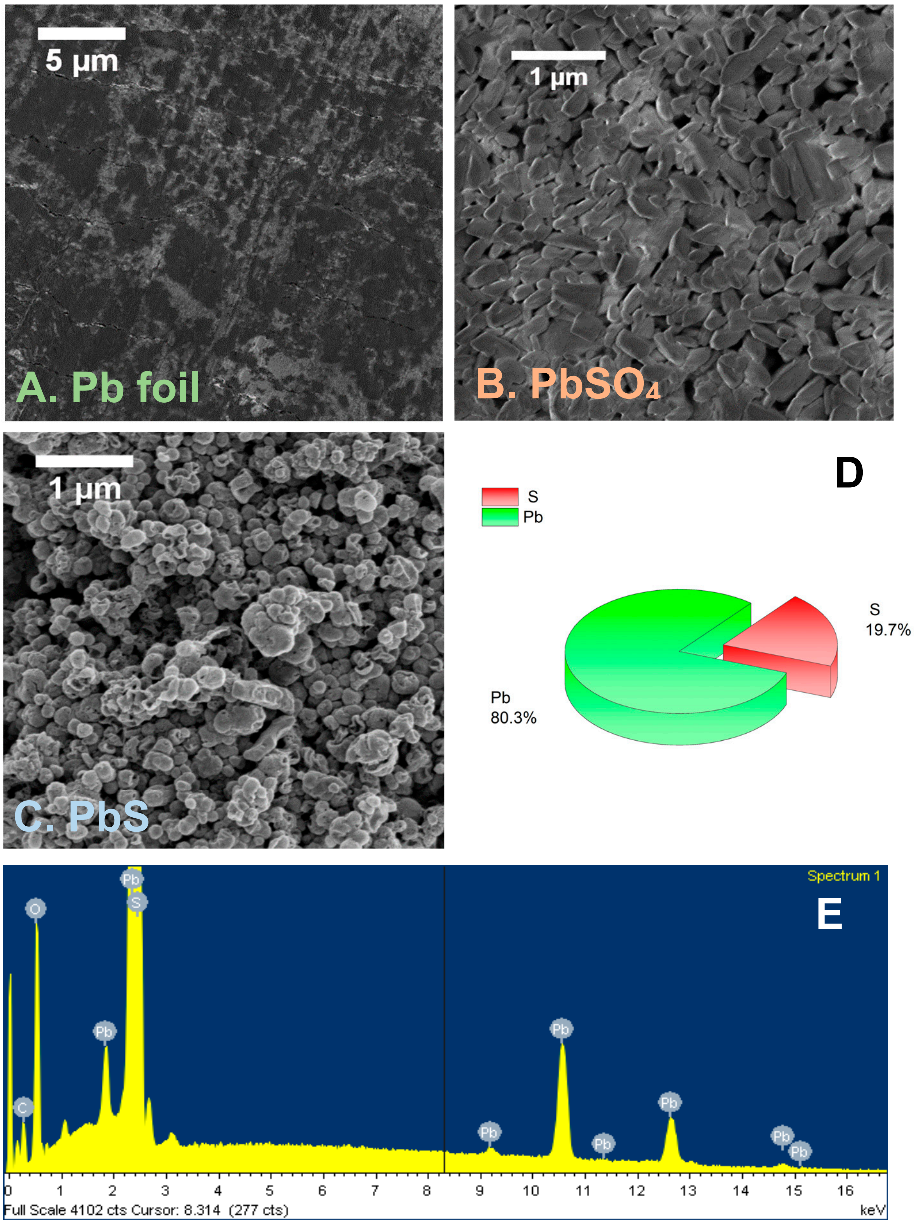

Figure S18. SEM images of the three stages in material as the originally purchased Pb foil (image **A**) is firstly converted to $PbSO_4$ (image **B**) and then to PbS (image **C**). To produce the desired PbS counter electrode. Graph **D** and **E** shows the elemental composition of the PbS film determined using EDX spectroscopy.

6.4. Cu_2S Counter Electrode

Cu_2S electrode was produced from a 0.1 mm foil of brass (alloy 260, Cu 68.5–71.5 %, Pb 0.07 % max, Fe 0.05 % max, Zn remainder) with the brass foil and produced electrode shown in Figure S19. The brass foil was firstly treated in a bath of concentrated HCl solution for 30 min. The function of this was to etch the Zn out of the brass foil surface, leaving a purely copper surface. This produced a reactive Cu surface, which was then converted to Cu_2S. This was carried out by immersing the electrode into an aqueous solution of 1 M Na_2S and 1 M S, for 10 min. Following this, the electrode was analysed with the use of SEM and EDX and is shown in Figure S20. SEM showed a huge change in the morphology from the smooth Brass electrode (Figure S20A) to Cu_2S with complex microstructure and morphology (Figure S20B). EDX was then utilised to determine the elemental composition of the electrode before (Figure S20C,E) and after treatment (Figure S20D,F). The elemental makeup of the brass foil was determined to be Zn 31.8% relative to Cu 68.2% before treatment, which was then shown to convert to Cu 64%, Zn 28.5%, and S 7.5% after treatment,

confirming the formation of a Cu_2S surface coating [15]. Brass foil was used instead of a purely copper due to the presence Zn, which helps to form a more stable support for the Cu_2S film.

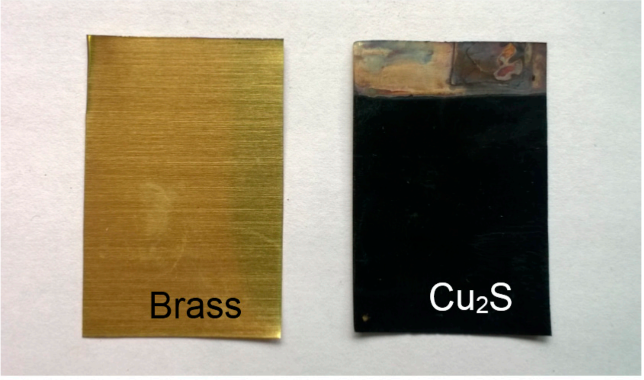

Figure S19. Photos of original brass foil and the produced Cu_2S coated foil produced from this.

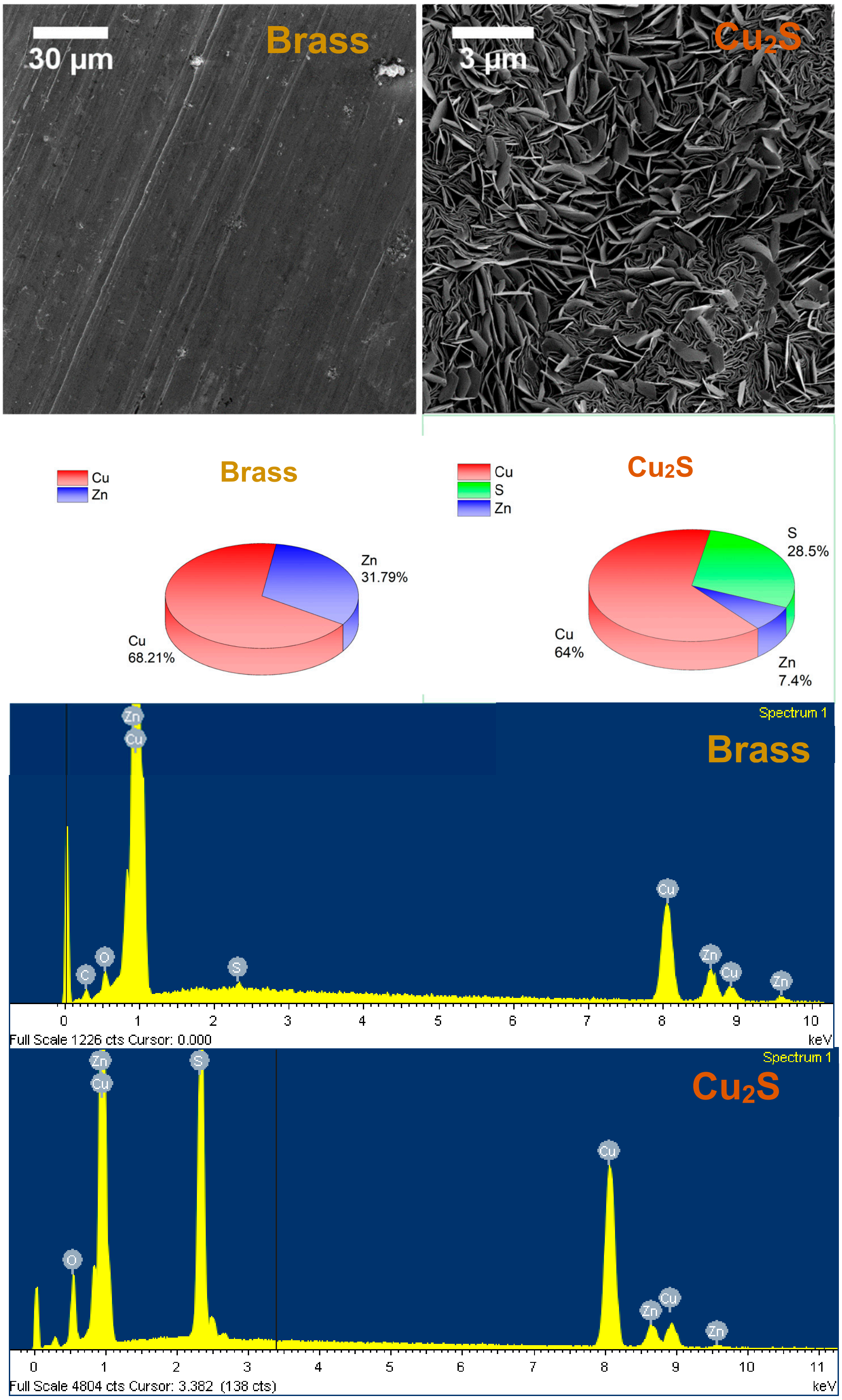

Figure S20. SEM images of brass foil before and after treatment to produce the Cu_2S electrode. The elemental composition of the foils was examined using EDX and compared to the original films described in the spectra and pie charts of both.

Table S1. This shows the result of open QDSSC cell testing under AM 1.5, 1 sun illumination, showing the resulting characteristics of the cells determined by measuring the IV response of the produced cells.

Cell Parameters	Cell 1 (CdSe QDs, 4.3 nm, OA capped, CE:PbS)	Cell 2 (CdSe QDs, 4.3 nm, OA capped, CE:Cu_2S)	Cell 3 (CdSe QDs, 5.1 nm, ODPA capped, CE:Cu_2S)
I_{sc} (mA/cm^2)	2.67	3.22	1.03
V_{oc} (V)	0.48	0.46	0.44
Fill factor (%)	29.82	33.84	38.15
Cell size (cm^2)	2	2	2
PCE (%)	0.38	0.501	0.172

7. Additional QDs Electrophoretic Deposition

7.1 EPD of Octadecylphosphonic acid capped CdSe QDs.

QDs were synthesised in the presence of octadecylamine and oleic acid meaning the ligand shell consisted of a mix of both ligands upon the surface. Deposition was carried out under the same conditions as described for oleic acid capped CdSe QDs. Following this photocurrent response was measured and is shown in Figure S16. The different ligand shell and conditions of synthesis meant that the QDs had to go through three cleaning cycles before deposition was possible from the DCM solution, with deposition occurring on the negative electrode. Unfortunately, the repeatability of deposition was moderate at best especially when attempting to deposit QDs of larger sizes, which regularly precipitated from solution under the applied voltage as opposed to sensitising the TiO_2 electrode. Therefore, despite these QDs showing excellent sensitisation when deposition did occur, further use of these ODA capped CdSe QDs was discontinued.

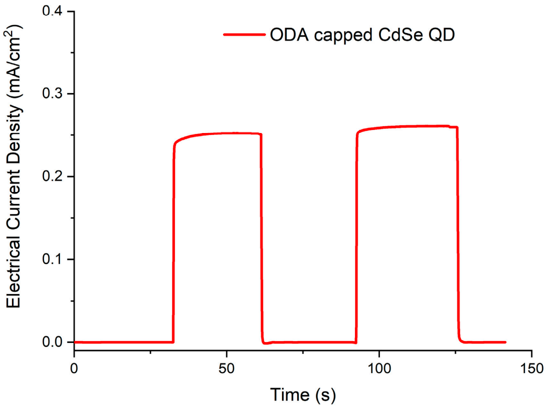

Figure S21. Photocurrent action response of ODA capped CdSe QDs showing a peak current of 0.25mA/cm^2 from 4.2 nm CdSe QDs.

7.2. EPD of Octadecylphosphonic Acid Capped CdSe QDs

For octadecylphosphonic (ODPA) capped CdSe QDs, it was found that due to the conditions of synthesis meant that even after numerous cleaning steps, involving dissolution in toluene, followed by precipitation due to addition of MeOH, an excess of ligands remained in solution. It was found that under these conditions the QDs could not be deposited by EPD upon a TiO_2 electrode from DCM whatsoever. Therefore, an additional cleaning step was included, which involved dissolving these QDs into hexane and centrifuging at 4000 rpm for 20 min, producing a stable solution of QDs with a deposit of excess ligands that was then separated and discarded. After this it became possible to

deposit the QDs, but deposition time had to be increased to 20–30 min depending on the composition of solution being used. Interestingly, that after depositing, ODPA capped CdSe QDs solution from a 20 mL solution of 5 × 10^{-6} M of CdSe QDs concentration, it was found that additional cycles of up to 8 depositions could be produced from the same solution while maintaining the same level of loading upon electrodes In addition, it was noted that no loss of deposition ability occurred due to prolonged dilution in DCM as was seen with oleic acid capped CdSe QDs. In overall the deposition was excellent and produced effective sensitization with UV-Vis absorption and photocurrent response given in Figure S17.

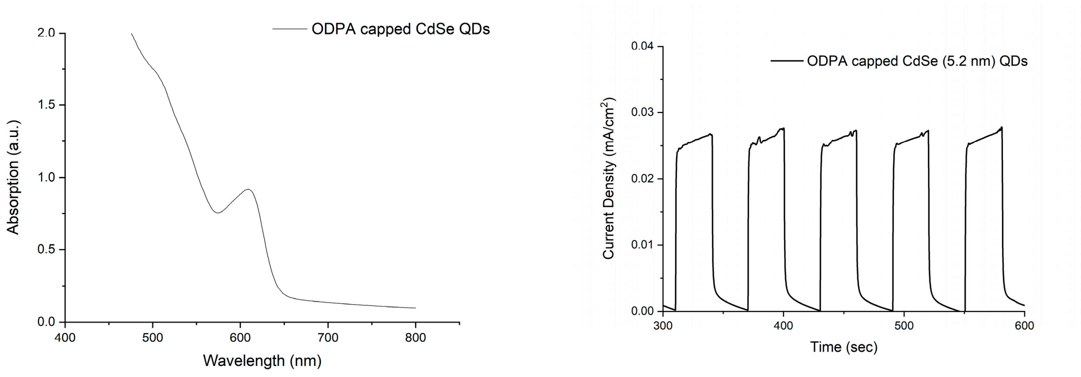

(**A**) (**B**)

Figure S22. UV-Vis absorption spectroscopy (**A**) and the resulting photocurrent action response (**B**) of ODPA capped CdSe QDs (5.2 nm) EPD deposited upon a TiO_2 electrode.

7.3. EPD of CdS QDs

CdS is synthesised with an oleic acid ligand surface. The resulting QDs are much smaller than CdSe, measuring between 2 to 3 nm in diameter. They were deposited using the same conditions as mentioned for CdSe, and the resulting UV-Vis spectra and photocurrent measurements are shown in Figure S23A,B respectively. Deposition took place upon the positive and negative electrode with stronger absorption taking place upon the negative electrode. This deposition pattern was ascribed to the ratio of 1:2, S:Cd used in the synthesis. Unfortunately, the deposition showed the same issues regarding repeatability raised using oleic acid capped CdSe QDs.

Due to the larger band gap of CdS QDs (band gap ≥3eV), these QDs are exclusively UV absorbers and therefore overlap strongly with the absorption of the TiO_2 electrode. They also therefore are poor sensitizers of TiO_2 only marginally widening the range of the visible spectrum the electrode can harvest. This is represented in the poor photocurrent responses produced of 0.0113 mA/cm^2 of the positive electrode and only 0.005 mA/cm^2 of the negative electrode.

SEM and EDX of the sensitised electrodes were then used to determine the elemental composition and the resulting loading of the TiO_2 film, shown in Figure S24. This confirms a linear distribution of CdS QDs throughout the electrode relative to depth (Figure S24A–C) and produced a loading of 1.75% of cadmium and 2.93% of sulphur relative to titanium present (Figure S24D,E), which compares poorly to the loading values achieved with oleic acid capped CdSe.

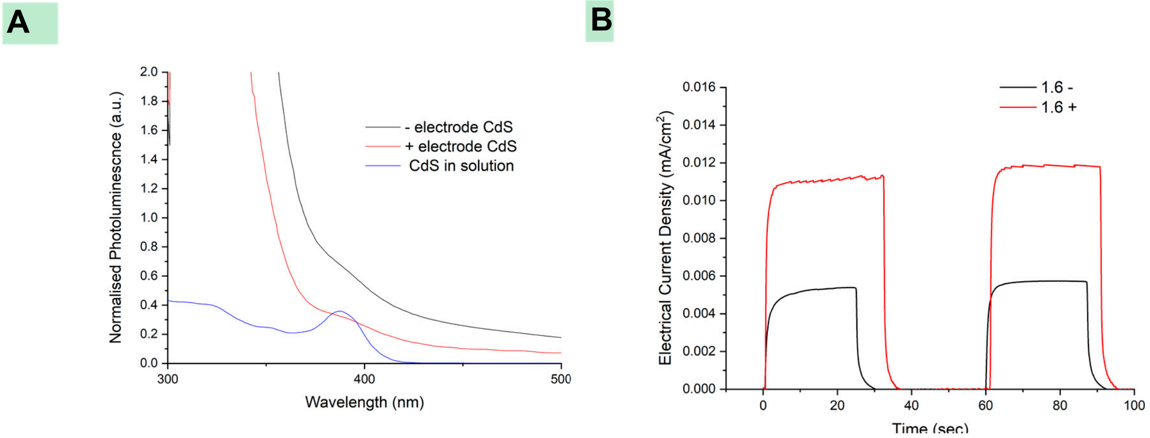

Figure S23. This shows the UV-vis absorption and the resulting photocurrent action response of a TiO_2-FTO electrode sensitized with 3 nm CdS QDs. (**A**) UV-Vis absorption shows the original QD spectra in solution and then the resulting spectra of the electrodes sensitized. The positive and the negative electrode both show depostion, with higher laoding taking place upon the postive elctrode. (**B**) Photocurrent action spectra shows a on-off response under illumination. 0.01127mA/cm^2, 0.0053mA/cm^2.

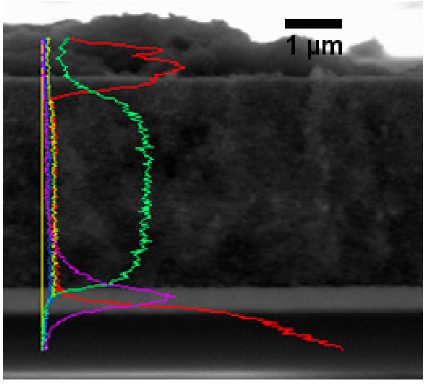

(**A**)

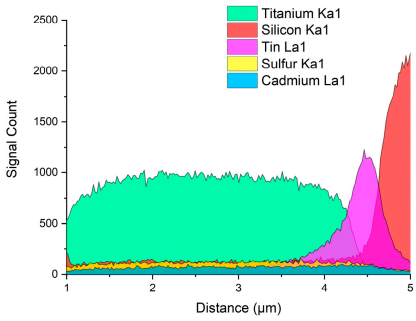

(**B**)

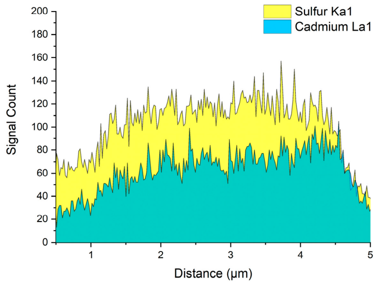

(**C**)

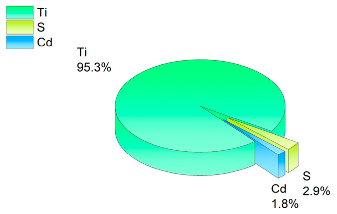

(**D**)

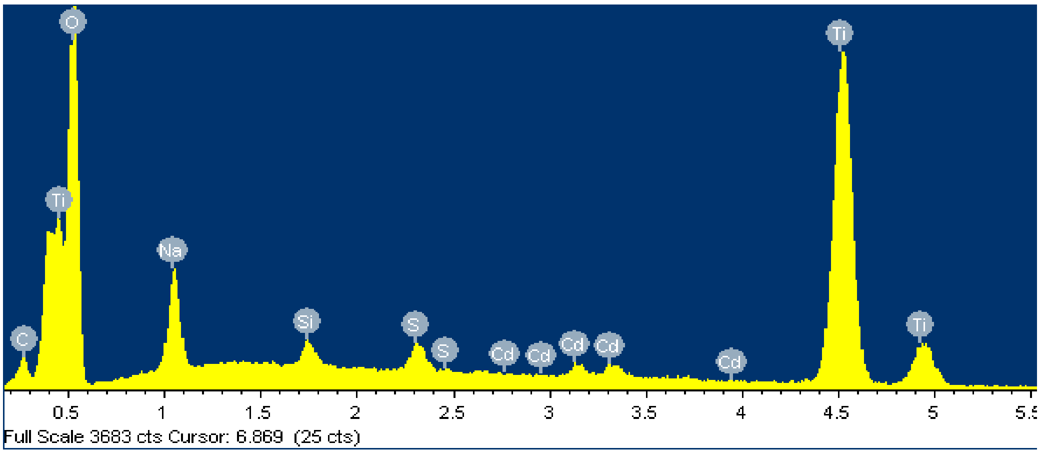

(**E**)

Figure S24. SEM and EDX of a CdS QD sensitised nanoporous 4 μm TiO_2 electrode with 3 nm CdS QDs. (**A**) Shows SEM of the side profile of TiO_2 electrode on FTO glass, with an EDX line profile overlaid on the image showing the relative abundance of different elements in this electrode. (**B**) EDX line profile of elemental composition while (**C**) is the EDX line profile just showing the distribution of just QD related materials, Cadmium and Sulphur across the electrode. Image **D** shows relative loading of QDs related materials in the TiO_2 electrode at depth of 2 μm relative to titanium while image E shows the EDX spectrum of the electrode at a depth of 2 μm in the TiO_2 electrode.

7.4. EPD of PbS QDs

PbS is a direct band gap semiconductor with a value of 0.37 eV in the bulk. Therefore, when synthesised as a QD, the bandgap can be tuned from NIR to IR values, making them a perfect candidate for producing a quantum dot with a band gap in the optimal range of the Shockley-Queisser limit, 1 eV to 1.5 eV. PbS QDs with diameters between 2.6 nm to 4.3 nm were therefore investigated.

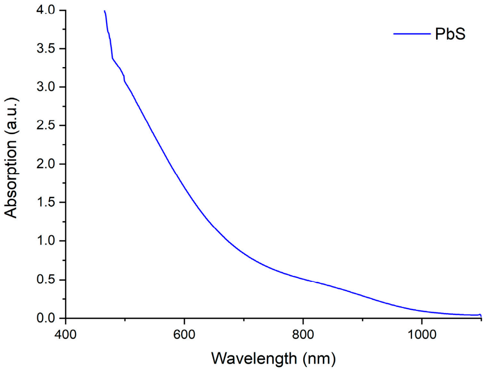

(**A**)

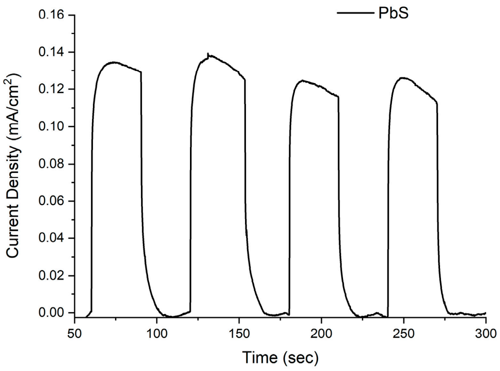

(**B**)

Figure S25. UV-Vis absorption spectra (**A**) and resulting photocurrent response (**B**) of PbS QD sensitised TiO_2 electrode. (**A**) Shows the resulting absorption of an electrophoretically deposited PbS (2.7 nm) QD upon a TiO_2 electrode. B shows the resulting photocurrent response under illumination, producing a peak current of 0.13 mA/cm^2.The same issues with deposition repeatability occurred with oleic acid capped PbS as was described with oleic acid capped CdSe QDs, presenting low repeatability, with solutions synthesised under identical conditions showing large variance with deposition rate. Instability of these solutions also contributed to this, with larger PbS QDs (diameter >4.0 nm) showing precipitation under the applied field. The majority of deposition occurred upon the positive electrode and is partially due to the 1:2 molar ratio of S:Pb precursors used in the PbS synthesis, producing Pb rich QDs.

The resulting electrodes were analysed with UV-Vis absorption spectroscopy and photocurrent action response measurements and is shown in Figure S25. The electrode showed a huge increase in absorption due to PbS deposition, producing absorption into the NIR due to the small band of the 2.7 nm QDs used. The photocurrent action response of the electrode produced a peak current of 0.13 mA/cm^2, which though substantial, is lower than expected due to the wide absorption of the electrode. The reason for the lower response than expected can be explained due to the light source used, a white LED ring, which shows a peak emission at 570 nm, and therefore is poorly matched to the absorption of the PbS sensitised electrode.

SEM and EDX were used to further analyse the sensitisation of TiO_2 electrodes, which is shown in Figure S26. The distribution of QDs was found to be near constant relative to depth of the TiO_2 (Figure S26A–C), as seen with other QDs examined previously, while the resulting loading achieved was very high, producing a loading of 7.26% Cd, 6.02% S relative to Ti present (Figure S26D,E).

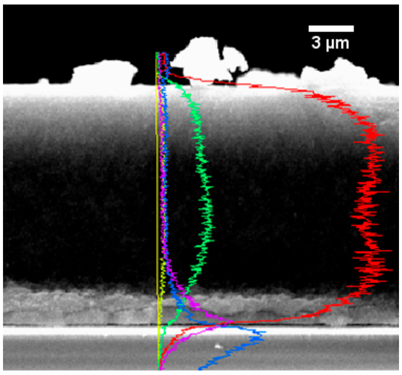

(**A**)

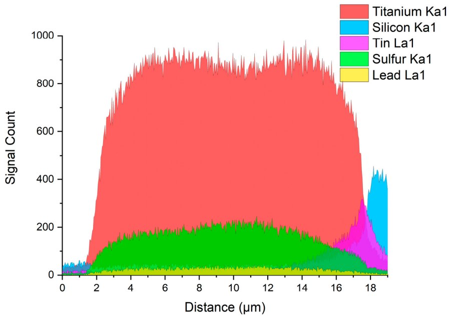

(**B**)

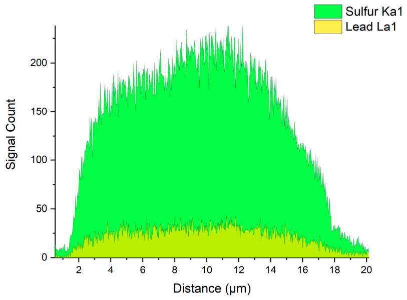

(**C**)

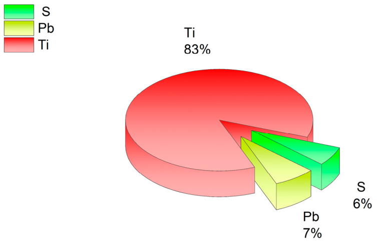

(**D**)

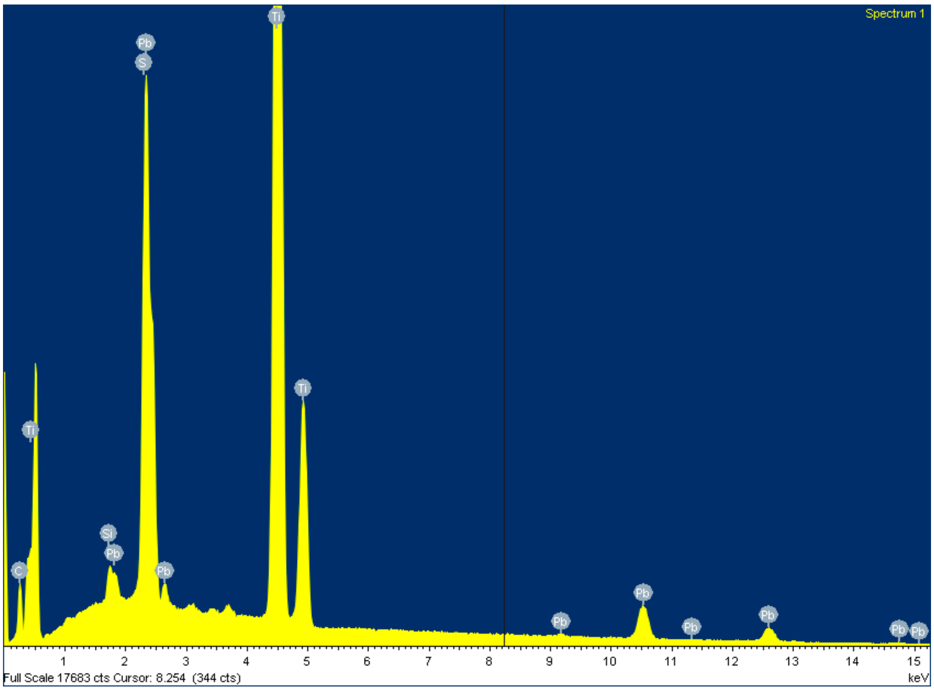

(**E**)

Figure S26. This shows the sensitisation of a nanoporous ~ 17-μm TiO_2 electrode with 2.7 nm PbS QDs. (**A**) Shows a SEM of the side profile of one of these electrodes with an EDX line profile overlaid on the image showing the relative abundance of different elements in this electrode. (**B**) EDX line profile of elemental composition. (**C**) EDX line profile show distribution of just QD materials across the electrode. (**D**) Elemental comparison of QDs in the TiO_2 electrode at depth of 7.5 μm. (**E**) EDX spectrum of the electrode at a depth of 7.5 μm in the TiO_2 electrode.

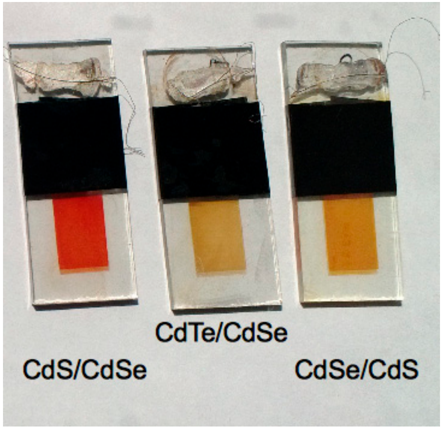

Figure S27. Photo of TiO_2 electrodes sensitised by CdS/CdSe QDs, CdTe/CdSe QDs, and CdSe/CdS QDs.

7.5. EPD of CdSe/CdS core-shell QDs

After testing three core QDs, it was decided to expand the study to core/shell QDs to understand the effects that these structures would have upon the photosensitisation and to deem if it was feasible to sensitise with these larger (in most cases) and more complex QD structures. Therefore, this was begun by investigating CdSe/CdS QD. This has a type I band structure meaning the exciton is confined to the inner CdSe QD core, and therefore have less interaction with surface states. This means that the exciton bears less chance of undergoing recombination through surface states or with the surrounding electrolyte, though it could also have the effect of retarding charge injection due to increased distance between the exciton and the TiO_2 surface. A CdSe/CdS QDs was formed using a

3.4 nm CdSe core and were deposited using EPD, and the resulting UV-Vis absorption is shown in Figure S28. Following this the photocurrent shown in the inset of Figure S28. The maximum current recorded was 0.05 mA/cm², which equates to a low response, which can be attributed to the poor loading achieved from the electrophoretic deposition as shown in the UV-Vis spectra. The QD loading was poor due to them being synthesised using a mix of oleylamine, octadecylamine and oleic acid as ligands, which strongly defined the behaviour under electrophoretic deposition, with QDs showing only partial deposition under the applied voltage, with deposition occurring only upon the negative electrode.

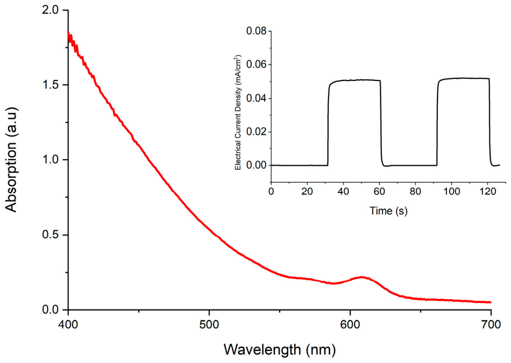

Figure S28. UV-Vis absorption spectra and photocurrent response (inset) of CdSe/CdS sensitised TiO_2 electrode. The total photocurrent produced was 0.05 mA/cm².

7.6. EPD of CdS/CdSe Core/Shell QDs

The second core-shell QD that was electrodeposited was CdS/CdSe, which is a reverse type I QD, meaning the exciton is more strongly confined to the surface of the QD, which has been reported to enable charge injection to occur more readily.[7] Therefore, it is also an interesting contrast to CdSe/CdS QDs already investigated. These QDs are synthesised using oleic acid as a capping ligand and so electrophoretic deposition was similar to other oleic acid capped QDs discussed. Due to the increased size of the QDs and the inherent challenge regarding concentration determination, the loading achieved of the TiO_2 electrode was not to the level achieved using oleic acid capped CdSe QDs. The film was analysed using UV-Vis absorption spectra and photocurrent response as shown in Figure S29. Even though total loading was suboptimal to light harvesting, the total current produced was much higher than expected, producing a current of 0.57 mA/cm² under illumination, indicating the effectiveness of this core shell structure to charge injection.

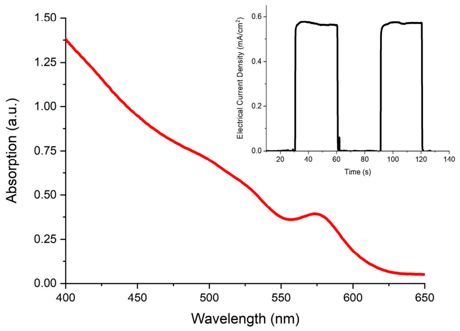

Figure S29. UV-Vis absorption spectra and photocurrent response (inset) of CdS/CdSe sensitized TiO_2 electrode, giving a photocurrent of 0.57mA/cm².

7.7. EPD of CdTe/CdSe Core/Shell QDs

The last form of core-shell nanostructures investigated was a CdTe/CdSe core-shell QDs. This core/shell structure produces a staggered band alignment between CdSe and CdTe, causing the band gap of the resulting QD to be smaller than either semiconductor. This also produces the effect that the exciton is separated across the QD, with the hole confined to the core, while the election is confined to the shell. The QDs were synthesised using oleic acid and a small amount of ODPA and produced QDs with a diameter of 5.5 nm. Electrophoretic deposition proved difficult with these QDs due to their ligand coating and larger size and therefore loading was not optimal. The resulting UV-Vis absorption achieved is shown in Figure S30 while the produced photocurrents are shown in the inset with maximum photocurrents of 0.316 mA/cm^2 recorded. Again, this electrode in fact outperformed expectations due to the pure loading of QDs achieved making it an interesting candidate for further investigation.

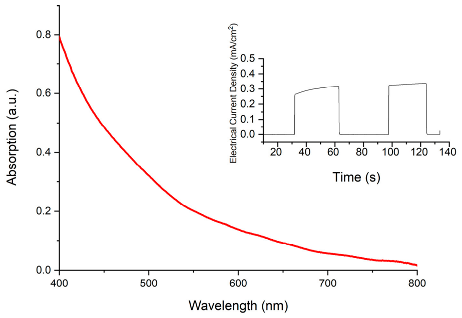

Figure S30. UV-Vis absorption spectra CdTe/CdSe QD sensitised TiO_2 electrode and photocurrent response (inset) of the resulting electrode measured under illumination producing a current of 0.316 mA/cm^2.

References

1. Yu, W.W.; Peng, X. Formation of high-quality CdS and other ii–vi semiconductor nanocrystals in noncoordinating solvents: Tunable reactivity of monomers. *Angew. Chem. Int. Ed.* **2002**, *41*, 2368-2371.
2. Ouyang, J.; Kuijper, J.; Brot, S.; Kingston, D.; Wu, X.; Leek, D.M.; Hu, M.Z.; Ripmeester, J.A.; Yu, K. Photoluminescent colloidal CdS nanocrystals with high quality via noninjection one-pot synthesis in 1-octadecene. *The Journal of Physical Chemistry C* **2009**, *113*, 7579-7593.
3. Bullen, C.R.; Mulvaney, P. Nucleation and growth kinetics of CdSe nanocrystals in octadecene. *Nano Lett.* **2004**, *4*, 2303-2307.
4. Carbone, L.; Nobile, C.; De Giorgi, M.; Sala, F.D.; Morello, G.; Pompa, P.; Hytch, M.; Snoeck, E.; Fiore, A.; Franchini, I.R., *et al.* Synthesis and micrometer-scale assembly of colloidal CdSe/CdS nanorods prepared by a seeded growth approach. *Nano Lett.* **2007**, *7*, 2942-2950.
5. Mahler, B.; Spinicelli, P.; Buil, S.; Quelin, X.; Hermier, J.P.; Dubertret, B. Towards non-blinking colloidal quantum dots. *Nat Mater* **2008**, *7*, 659-664.
6. Liu, T.Y.; Li, M.J.; Ouyang, J.Y.; Zaman, M.B.; Wang, R.B.; Wu, X.H.; Yeh, C.S.; Lin, Q.; Yang, B.; Yu, K. Non-injection and low-temperature approach to colloidal photoluminescent PbS nanocrystals with narrow bandwidth. *Journal of Physical Chemistry C* **2009**, *113*, 2301-2308.
7. Pan, Z.; Zhang, H.; Cheng, K.; Hou, Y.; Hua, J.; Zhong, X. Highly efficient inverted type-i CdS/CdSe core/shell structure qd-sensitized solar cells. *Acs Nano* **2012**, *6*, 3982-3991.

8. Cai, X.; Mirafzal, H.; Nguyen, K.; Leppert, V.; Kelley, D.F. Spectroscopy of CdTe/CdSe type-ii nanostructures: Morphology, lattice mismatch, and band-bowing effects. *The Journal of Physical Chemistry C* **2012**, *116*, 8118-8127.
9. Adachi, M.M.; Labelle, A.J.; Thon, S.M.; Lan, X.; Hoogland, S.; Sargent, E.H. Broadband solar absorption enhancement via periodic nanostructuring of electrodes. *Scientific Reports* **2013**, *3*, 6.
10. Pattantyus-Abraham, A.G.; Kramer, I.J.; Barkhouse, A.R.; Wang, X.H.; Konstantatos, G.; Debnath, R.; Levina, L.; Raabe, I.; Nazeeruddin, M.K.; Grätzel, M., *et al.* Depleted-heterojunction colloidal quantum dot solar cells. *ACS Nano* **2010**, *4*, 3374-3380.
11. Besra, L.; Liu, M. A review on fundamentals and applications of electrophoretic deposition (epd). *Progress in Materials Science* **2007**, *52*, 1-61.
12. Biesheuvel, P.M.; Verweij, H. Theory of cast formation in electrophoretic deposition. *Journal of the American Ceramic Society* **1999**, *82*, 1451-1455.
13. Hamaker, H.C. Formation of a deposit by electrophoresis. *Transactions of the Faraday Society* **1940**, *35*, 279-287.
14. Tachan, Z.; Shalom, M.; Hod, I.; Ruhle, S.; Tirosh, S.; Zaban, A. PbS as a highly catalytic counter electrode for polysulfide-based quantum dot solar cells. *J. Phys. Chem. C* **2011**, *115*, 6162-6166.
15. Lee, J.-W.; Son, D.-Y.; Ahn, T.K.; Shin, H.-W.; Kim, I.Y.; Hwang, S.-J.; Ko, M.J.; Sul, S.; Han, H.; Park, N.-G. Quantum-dot-sensitized solar cell with unprecedentedly high photocurrent. *Sci. Rep.* **2013**, *3*.

© 2019 by the authors. Submitted for possible open access publication under the terms and conditions of the Creative Commons Attribution (CC BY) license (http://creativecommons.org/licenses/by/4.0/).
